# Differential role of internet-based targeted persuasive advertising versus mass advertising on firms with unique qualities in an anchoring perspective

**DOI:** 10.1371/journal.pone.0325552

**Published:** 2025-07-01

**Authors:** Zhao Jiang, Wu Dan, Yue Lin-Jun

**Affiliations:** 1 School of Business, Shaoxing University, Shaoxing, Zhejiang, China; 2 Shaoxing Local Legislative Research Center, Shaoxing, Zhejiang, China,; 3 Academy of Art, Shaoxing University, Shaoxing, Zhejiang, China; Duquesne University Palumbo Donahue School of Business, UNITED STATES OF AMERICA

## Abstract

A firm’s primary objective is to enhance its products’ perceived quality through persuasive advertising techniques, thereby influencing consumer purchase decisions. This research paper delves into the effects of different forms of persuasive advertising, specifically mass and Internet-based targeted persuasive advertising, on enhancing perceived product qualities within a competitive duopoly. The analysis takes into consideration a vertical differentiation model. The findings of this study demonstrate that both firms, each producing products with distinctive qualities, can augment the perceived quality of their offerings through the application of persuasive advertising strategies. Notably, a low-quality firm consistently adjusts its perceived quality to a fixed ratio of 4/7, leading to the anchoring effect on perceived quality. Meanwhile, it is observed that the firm manufacturing lower-quality products tend to rely more on mass persuasive advertising in comparison to its rival firm. Furthermore, targeted advertising through online channels proves to be advantageous in reaching a specific population that highly values the price-to-quality ratio. Specifically, a firm with superior quality that engages in Internet-based targeted persuasive advertising stands to gain more incredible benefits than its rival relying on mass persuasive advertising. These findings shed light on the distinct impacts of various persuasive advertising approaches on competitive firms with unique qualities. Essentially, it underscores the significance of strategic advertising decisions in effectively influencing consumers’ perceived product qualities and gaining a competitive edge in the market.

## 1 Introduction

Advertising is widely acknowledged as a pivotal marketing strategy for firms, encompassing two primary types: informative and persuasive. Informative advertising, exemplified by specific product information like locations, prices, and features, substantially enhances market performance. Moreover, this type of advertising can play a critical role in building trust and credibility, as it provides consumers with transparent and valuable insights into the products available on the market. It can also increase consumer awareness, enabling more informed decision-making processes. Conversely, although persuasive advertising cannot directly alter product quality, it boosts perceived product value by cultivating a distinctive brand persona. Through persuasive advertising, consumer perceptions of product quality can be shaped, influencing purchasing decisions upon receipt of advertising messages. Given advertising’s multifaceted nature and the context of product differentiation within an oligopoly, advertising is inherently perceived as persuasive (Aylsworth, 2022) [1]. By utilizing persuasive advertising, a firm can convey differentiated product information compared to competitors, thereby persuading consumers to opt for its offerings. This type of advertising holds the potential for reshaping consumer preferences by fostering subjective differentiation and stimulating purchases that might not have occurred otherwise. Targeted advertising relates to industry relevance. For instance, E-commerce giants like Amazon and Alibaba utilize targeted advertising to personalize the shopping experience for millions of users based on their browsing history and purchase behavior. By understanding the effectiveness of targeted versus mass advertising, smaller e-commerce firms can make informed decisions about their advertising investments to compete more effectively in the market. Otherwise, car manufacturers often segment their advertising strategies based on product quality. Luxury brands like Tesla and BMW might benefit more from targeted advertising highlighting their superior features to a niche audience. In contrast, mass-market brands like Toyota might opt for broader advertising campaigns.

Additionally, targeted advertising is also used in consumer electronics. Tech companies like Apple and Samsung use targeted advertising to showcase unique product qualities to tech-savvy consumers while also employing mass advertising to build brand awareness among a wider audience. The rapid evolution of digital technology has significantly transformed the advertising landscape, leading to the emergence of highly targeted and data-driven advertising methods. Businesses today face the challenge of selecting the most effective advertising strategies to enhance the perceived quality of their products and ultimately influence consumer purchase decisions. The primary objective of this research is to investigate the comparative effects of mass persuasive advertising, and Internet-based targeted persuasive advertising on firms producing products with distinct quality levels within a competitive duopoly.

## 2 Literature review

### 2.1 Mass advertising in the digital age

Product differentiation can be categorized into two specific types. The first is horizontal differentiation, evident when consumers have distinct preferences for specific product attributes. For instance, consider two competing beverage firms differing solely in geographic locations within a city. In such cases, consumers typically favor products with more convenient locations—a concept akin to spatial differentiation or the Hotelling model (Nakagawa, 2023) [2]. Horizontal differentiation can also extend to product variations based on style, taste, or other attributes, offering consumers a more comprehensive array of choices suited to their tastes and preferences. The second type is vertical differentiation, where consumers universally agree on preferred attributes and the broader preference order. An illustrative example of vertical differentiation occurs when products vary in quality, with consumers universally preferring a smaller and more powerful computer over a larger, less powerful one, holding prices constant. Vertical differentiation concerns quality distinctions and the pursuit of offering superior products compared to competitors. In contrast, horizontal differentiation aims to create comparable products with unique characteristics to cater to diverse consumer preferences. This approach highlights product features such as advanced technology, durability, and design. Furthermore, advertising can be leveraged to strategically position a firm’s products within the market, whether through highlighting unique selling propositions or leveraging celebrity endorsements to enhance brand image. By effectively combining informative and persuasive advertising techniques, firms can maximize their market reach and appeal to a broader consumer base. As the advertising landscape evolves with emerging technologies, such as data analytics and AI-driven targeting, firms must remain agile and innovative in their advertising strategies to stay competitive and resonate with consumers.

While mass advertising can create broad awareness, it often fails to deliver personalized messages that resonate with individual consumer preferences. Studies by Thomos [3] suggest that mass advertising may be less effective in markets characterized by high product differentiation, as it cannot effectively communicate unique product qualities to specific consumer segments. Previous studies have focused on analyzing particular types of advertising and product differentiation within a competitive context. For example, Grossman and Shapiro (1984) revealed the impact of informative advertising on price competition in an oligopoly market characterized by horizontal differentiation [4]. Milgom and Roberts (1986) demonstrated how advertising can serve as a signal of quality for high-quality producers [5]. Similarly, Hallagan and Joerding (1983) examined the effects of persuasive advertising in a monopolistic market [6]. Building on this research, Lee (1997) investigated the influence of persuasive advertising on socialization [7]. Tremblay V and Martins-Filho (2001) utilized a vertical model to explore the relationship between persuasive advertising and price competition [8]. Albrecht’s (2023) search-theoretic model of imperfect competition in product markets where sellers make an ex-ante investment in the quality of their product variety [9]. Similarly, Liu Q and Serfes (2005) demonstrated that if the cost of information falls below a certain threshold, only the high-quality firm will invest in it and offer targeted promotions [10]. In a previous study, Lauga (2011) demonstrated that while firms attempt to manage competition through product positioning, the effectiveness of their differentiation strategies depends crucially on the cost associated with providing higher quality [11]. Moreover, Liu M et al. (2012) examined the impact of advertising support on the success of online services and established that higher ad revenue rates may result in lower service prices [12]. Their findings underscore the significance of advertising revenues in the context of vertical differentiation and offer strategic guidance for monetizing online services. Finally, Guan H et al. (2023) explored a duopoly scenario and the manufacturer’s strategic decision in a peer-to-peer sharing platform with quality differentiation [13].

Mass advertising campaigns are often costly and may not yield a proportional return on investment, especially when targeting a diverse audience with varying preferences. This raises questions about the cost-effectiveness of mass advertising in achieving optimal market performance for firms with distinct product qualities. Our research is situated within the relevant literature on targeted advertising. Hernandez-Garcia (1997) was among the first to examine the impact of informative targeted advertising on firm profitability and social welfare within a monopolistic framework [14]. Subsequently, Iyer (2005) demonstrated that targeted advertising has more value than targeted pricing [15]. Johnson JP (2013) provided evidence that enhanced targeting can benefit firms even in the presence of consumer ad avoidance, although such improvements may have negative consequences for consumers [16]. Similarly, Esteves RB and Resende (2016) verified that through targeted advertising and price discrimination, firms tend to advertise more intensively within their targeted segments [17]. Recent studies by Zhao J. and Dan W. (2022) have shown that implementing imperfect targeted advertising with moderate targeting precision can facilitate the entry of new competitive rivals and yield overall market efficiency [18]. This effect is particularly pronounced when the weaker segment is desirable, and advertising costs are sufficiently high. However, it is worth noting that certain studies have questioned the comparative effectiveness of targeted advertising versus mass advertising.

### 2.2 Effectiveness of targeted advertising

Gal-Or et al. (2006) introduced a two-dimensional parameter to assess targeting quality and demonstrated that increased recognition could intensify price competition, thereby reducing firm profit [19]. Expanding on this line of research, Elhadj-Ben Brahim et al. (2011) established that a firm’s equilibrium profit may be lower when ideal differentiation among consumer types is achievable [20]. However, it is worth noting that these studies primarily focused on informative targeted advertising. Esteban L (2007) demonstrated that targeting can lead to permanent market fragmentation, resulting in local monopolies within a vertical differentiation model [21]. Their findings align closely with those of Iyer et al. (2005) [15]. Similarly, Zhang (2018) discovered that targeted advertising can adversely impact both firms, even when it is more cost-efficient than mass advertising, due to the well-known dilemma of the prisoner’s dilemma [22].

Nevertheless, traditional advertising operational models are insufficient to meet the current demands of a growing e-commerce market. During the past few decades, this has led to the increasing integration of artificial intelligence (AI) as a core technology in the advertising and media industries. AI enables rapid and effective implementation. Advertisers and e-commerce platforms utilize AI technologies to improve advertising efficiency and cater to market demands (Qin and Jiang, 2019) [23]. However, this has resulted in an ongoing debate on the value of different types of targeting information, the incentives of ad networks to engage in behavioral targeting, and the role of regulation (Rafieian, 2021) [24]. In a recent study, Zhang et al. (2021) proposed an innovative multimodal-based marketing intent analysis scheme to assess the embedded marketing intent within multimodal content [25]. They validated the potential of such an approach in enhancing the perceived value of products. Similarly, Li et al. (2023) investigated the optimal advertising strategy for two heterogeneous retailers and found that when consumers’ anticipated targeted effect is positive, both retailers benefit from targeted advertising, with the weaker retailer deriving more significant benefits than the stronger one [26].

Additionally, while AI has already automated the media buying and delivery processes, creative advertising still requires substantial human effort (Li H, 2019) [27]. This study aims to investigate the circumstances under which firms competing in a vertical market with distinct qualities choose persuasive advertising to enhance product quality. Furthermore, we strive to determine which firm benefits more from adopting persuasive advertising and explore whether internet-based targeted persuasive advertising is consistently more advantageous than mass persuasive advertising. Human judgment is often influenced by salient norms and standards. One prominent effect is the anchoring effect, which refers to the tendency for numeric estimates to assimilate towards previously considered standards. Extensive research has demonstrated the influence of judgmental anchoring in various domains, such as general knowledge, legal decision-making, pricing choices, and negotiation. Businesses also leverage the “anchor effect” when introducing new products. For instance, consumers may hesitate to accept a high price if a new high-end cosmetic is positioned amidst low-cost goods, even with superior quality. Conversely, its higher cost may be readily accepted if the same high-end cosmetic is strategically placed alongside other reputable brands. Persuasive advertising can be utilized to improve the perceived quality of a product. The anchoring effect, as described by Wujin C H U(2024), refers to the cognitive bias where individuals rely heavily on an initial piece of information (the anchor) when making subsequent judgments [28]. While this effect is well-documented in various domains, its application in the context of advertising strategies for firms with different product qualities remains underexplored.

### 2.3 Anchoring in marketing contexts

The increasing availability of consumer data and advancements in machine learning algorithms have made Internet-based targeted advertising more accessible and practical. According to eMarketer, digital ad spending will account for over 60% of total media ad spending by 2024. This shift underscores the growing importance of understanding how targeted advertising can influence consumer perceptions and firm performance, particularly for firms with distinct product qualities. Hence, we are interested in determining how persuasive advertising shapes perceived quality in the market and exploring the presence of anchoring effects in a competitive market when firms employ persuasive advertising strategies. Understanding the differential impacts of these advertising strategies holds immense practical significance for businesses aiming to optimize their marketing investments. Specifically, Internet-based targeted persuasive advertising leverages sophisticated algorithms and data analytics to deliver highly personalized advertisements, potentially enhancing marketing efforts’ relevance and effectiveness. In contrast, mass persuasive advertising reaches a broader audience but lacks the precision to target specific consumer segments. For instance, Zhang and Wedel (2009) call for more research into the psychological mechanisms behind personalized advertising, which aligns with our focus on the anchoring perspective [29]. Sharakhina L (2024) considered that AI analytics provides the ability to track the level of audience engagement in perceiving video content, which helps to improve communication effectiveness [30]. Sharma et al.(2024) reveal that advertising value more substantially affects purchase intention than attitude toward advertising. Advertising irritation is a strong negative moderator that significantly reduces overall advertising effectiveness [31]. Additionally, there’s still a lack of focus on how anchoring effects interact with advertising strategies.

Previous studies have extensively explored the effects of mass and targeted advertising on consumer behavior and firm performance. However, a significant gap remains in understanding how these advertising strategies influence firms with products of different quality levels within a competitive duopoly. Existing research often treats advertising strategies in isolation without considering the interplay between mass and targeted advertising in vertical differentiation models. Kircher and Foerderer (2024) prove that consumers and policymakers should know that targeted advertising plays a crucial role in app development and can use their estimates to design policies [32]. What remains unexplored is the comparative effectiveness of mass versus targeted advertising for firms with unique product qualities and how these strategies influence perceived product quality and consumer purchase decisions. Likewise, Jiang Z et al.(2024) provide the role of targeted advertising in a distinct channel and point out the marketing strategy of targeting [33]. Specifically, the anchoring effect, where previously considered standards influence consumer judgments, has not been thoroughly examined in the context of these advertising strategies. To address this gap, our research aims to answer the following question: How do mass persuasive advertising and Internet-based targeted persuasive advertising differentially impact the perceived quality and market performance of firms producing products with distinct quality levels within a competitive duopoly, considering the anchoring effect on perceived quality?

### 2.4 Research motivation and overview

The motivation for this study stems from the increasing complexity of competitive markets, particularly in duopolistic settings where firms employ product differentiation and persuasive advertising to influence consumer behavior. In such markets, the perceived quality of a product plays a critical role in shaping consumer purchase decisions, often outweighing the actual quality of the product. Understanding how different advertising strategies—specifically mass and Internet-based targeted persuasive advertising—affect perceived quality is essential for firms seeking a competitive edge.

Traditional advertising models often focus on the direct impact of advertising on sales or brand awareness. Still, they rarely explore the nuanced relationship between advertising strategies and perceived product quality, especially in vertically differentiated markets. This gap in the literature is particularly relevant in the digital age, where Internet-based targeted advertising has emerged as a powerful tool for reaching specific consumer segments. This study aims to provide actionable insights for firms operating in competitive duopolies by examining how these advertising strategies influence perceived quality.

Additionally, the study is motivated by the need to understand the anchoring effect. In this phenomenon, a low-quality firm adjusts its perceived quality to a fixed ratio of the high-quality firm’s standard. This effect has significant implications for competitive dynamics, shaping how firms position their products and allocate resources to advertising. By exploring the interplay between advertising strategies, vertical differentiation, and the anchoring effect, this study seeks to contribute to both theoretical and practical understandings of market competition.

### 2.5 Research methods

The research adopts a vertical differentiation model to analyze the competitive dynamics between two firms producing products of distinct quality levels. This model is particularly suited for studying duopolistic markets, as it allows for examining how firms with different quality levels compete for market share. The study focuses on the role of persuasive advertising in enhancing perceived product quality, distinguishing between mass persuasive advertising and Internet-based targeted persuasive advertising.

The approach is grounded in established theories such as the Hierarchy-of-Effects Model, the Elaboration Likelihood Model, and the Vertical Differentiation Theory. These frameworks provide a foundation for understanding how advertising influences consumer perceptions and decision-making processes. The study also incorporates the concept of the anchoring effect, which is used to explain how initial quality perceptions shape subsequent consumer judgments.

The study employs a combination of theoretical modeling and empirical analysis to explore these dynamics. The theoretical model examines firms’ strategic decisions regarding advertising investments and their impact on perceived quality. The empirical analysis focuses on real-world scenarios, using case studies and market data to validate the theoretical findings.

The study utilizes a vertical differentiation model to analyze the competitive behavior of two firms in a duopolistic market. The model assumes that one firm produces a high-quality product while the other produces a low-quality product. Both firms employ persuasive advertising strategies to enhance the perceived quality of their products and influence consumer purchase decisions. The study examines two primary forms of persuasive advertising: mass and Internet-based targeted persuasive advertising. Its broad reach and generic messaging characterize mass advertising, while targeted advertising focuses on specific consumer segments based on their preferences and behaviors. The model incorporates the cost of advertising, the transmission cost of reaching consumers, and the impact of advertising on perceived quality.

The analysis considers the anchoring effect, where the low-quality firm adjusts its perceived quality to a fixed ratio (4/7) of the high-quality firm’s standard. This effect explains how firms position their products and allocate resources to advertising. The study also explores the conditions under which firms engage in mass or targeted advertising, considering each strategy’s cost-benefit trade-offs.

The study employs empirical methods to validate the theoretical findings, including case studies and market data analysis. These methods provide real-world evidence of how advertising strategies influence perceived quality and competitive outcomes. The study also uses simulations to test the robustness of the model and explore alternative scenarios.

The remainder of the paper is organized as follows: Section 2 establishes the benchmark model without advertising, Section 3 analyzes the effects of mass persuasive advertising on firm equilibrium profit and compares it to the benchmark model, Section 4 extends the model to incorporate internet-based targeted persuasive advertising and compares the effects of persuasive targeted advertising versus persuasive mass advertising on firm equilibrium profit, and Section 5 presents the potential impact of persuasive advertising on firms with distinct qualities before concluding the paper.

## 3 Benchmark model

### 3.1 Assumptions of the model

Assume two firms (indexed by 1 and 2) compete in a market, and each firm produces a single product, defined as brand one and brand two. Strategic variables include price, advertising, and product quality. Otherwise, quality is indexed by the parameter s . Both firms make the product with distinct attributes indexed by the parameters. A higher value s indicates a higher level of quality,$s\in \left[s_{L},s_{H}\right]$, $s_{L},s_{H}\in R$, and $s_{H}>s_{L}>0$. The parameters $s_{L}$ and $s_{H}$ are endogenous variables, which means firm L produces low-quality products, and firm H produces high-quality ones. To simplify the analysis, we assume both variable and fixed production costs to be zero (Jungbauer T, 2024) [34]. However, it is worth noting that even when fixed production costs are considered, the conclusion remains valid. In this context, consumers are presented with a finite selection of “price-quality” alternatives offered by the competing firms in the industry. It is essential to recognize that, in many economic decisions, consumers’ choice of quality is equally necessary as their consideration of quantity.

Following Hess [35], consumers have heterogeneous preferences that are represented by a taste parameter θ. Tastes are assumed to be distributed uniformly over the interval [0,1]. Each θ -type of consumer maximizes the following utility function $\left(U_{i}(\theta )\right)$ for the brand i. The first equation defines the utility function if the preference level exceeds the price-quality ratio. However, the utility function is zero if the preference level is equal to or greater than the price-quality ratio.


$$U_{i}(\theta )=\{\begin{array}{c} {}*20l\theta s_{i}-p_{i},for\begin{array}{cc} {}*20r & \;\theta >p_{i}/s_{i} \end{array}\\ 0,for\begin{array}{cc} {}*20l\begin{array}{cc} {}*20r\begin{array}{cc} {}*20r & \end{array} & \end{array} & \theta \leq p_{i}/s_{i} \end{array} \end{array}$$ (1)

In this context, the parameter $p_{i}$ represents the price of the product while $s_{i}$ representing the level of quality (or service) associated with a particular brand i. Unlike horizontal differentiation, where consumers have varying preferences for different product attributes, in this case, all consumers prefer a higher quality product over a lower quality product when sold at the same price. Additionally, a consumer with a higher taste θ will exhibit a greater preference for quality and demonstrate higher loyalty towards the brand offering superior quality, all else being equal. As a result, this specification incorporates elements of vertical product differentiation and consumer brand loyalty.

We assume that there exists a marginal consumer with a taste parameter $\theta_{m}$, who exhibits indifference when choosing between purchasing the product from firm H or firm L. That is the equation of $\theta_{m}s_{H}-p_{H}=\theta_{m}s_{L}-p_{L}$, which implies that $\theta_{m}=(p_{H}-p_{L})/(s_{H}-s_{L})$. Thus, the value $\theta_{m}$ can be considered the quality-adjusted premium the marginal consumer is willing to pay for product H.

### 3.2 Advertising and game sequence

Brands *i* and *j* are considered to be of similar quality, although persuasive advertising has the potential to influence consumers’ perceptions of quality. In this context, advertising costs encompass two components: the production cost, which enhances the consumer’s perception of quality, and the transmission cost, which involves disseminating the advertising information to different consumers. In the case of mass advertising, the production cost $\left(A_{x}\right)$ exhibits a monotonically increasing and convex relationship. Which means $(A_{x}{)}^{\prime }>0$, $(A_{x}{)}^{\prime \prime }>0$, and $A_{x}(0)=0$. The production cost parameter of advertising considered being k, and consumers’ improved perception of the perceived quality of the advertising is considered as the following $a_{x}$. That means the production cost of persuasive advertising can be defined as the following expression $A_{x}=\frac{k}{2}a_{x}^{2}$. However, the transmission cost of advertising is linear to the amounts of consumers (q), which is considered being $A_{y}=\lambda q$. Note that the parameter λ represents the propagation coefficient, which determines the effectiveness of advertising transmission. The distinction between mass advertising and targeted advertising lies in the ability to specifically target objective consumers based on their behaviors. Unlike mass advertising, which can be disseminated to all consumers through mass media channels, targeted advertising leverages artificial intelligence (AI) technology to identify specific consumers based on their past purchasing histories, which enables marketers to send internet-based, personalized advertisements to the identified consumers directly (Kietzmann et al., 2018) [36]. We assume that the consumers’ demand for the firm depends upon it $\theta_{m},p_{H},p_{L},s_{H},s_{L}$. Consumers have unit demands, and the market is large enough, with a total need of one. The firm’s profit is denoted as $\pi_{i}$, whereas the variable $q_{i}$ is the firm i ’s sales volume (i=H,L).

Product Quality (q): $q_{H}$: Quality level of the high-quality firm’s product; $q_{L}$: Quality level of the low-quality firm’s product. The quality levels are determined based on market research and consumer surveys, ensuring that the high-quality and low-quality designations are empirically justified. Perceived Quality (PQ) $PQ_{H}$: Perceived quality of the high-quality firm’s product after advertising. $PQ_{L}$: Perceived quality of the low-quality firm’s product after advertising. Perceived quality is measured using consumer perception surveys administered before and after exposure to the advertising campaigns. Participants are randomly assigned to view mass persuasive or Internet-based targeted advertising for high-quality and low-quality products. The experiment simulates a competitive market environment, replicating real-world conditions.

Both firms engage in a three-stage game to maximize their profits. In the first stage, firms with unique product qualities strategically determine the optimal pricing. In the second stage, firms simultaneously make decisions regarding product quality and persuasive advertising, engaging in fierce competition. At each sub-game, firms have access to information regarding the outcomes of the previous stage. With persuasive advertising to influence consumer behavior and encourage purchases, firms have ample time to develop and execute their marketing campaigns. This allows them to establish effective marketing strategies before engaging in the quality and pricing stage games. Finally, in the last step, consumers typically purchase products from firms based on their unique attributes. We aim to solve the sub-game for perfect equilibrium in this game.

The paper introduces a research model based on two types of firms operating within a competitive duopoly, which can be interpreted through game theory, specifically non-cooperative games. The concept of Nash equilibrium, a cornerstone of non-cooperative game theory, is particularly relevant to this study as it provides a framework for analyzing the strategic interactions between the two firms. Under the Nash equilibrium, each firm’s strategy is optimal given the strategies of the other firm, and no firm has an incentive to deviate from its chosen strategy unilaterally.

In the context of this study, the Nash equilibrium would offer a more rigorous and meaningful outcome by formalizing the strategic decisions of both firms regarding their advertising investments and their impact on perceived product quality. Specifically, the Nash equilibrium would allow us to analyze how the firms’ choices of mass persuasive advertising versus Internet-based targeted persuasive advertising are influenced by their mutual expectations of each other’s actions. For instance, the high-quality firm’s decision to engage in targeted advertising and the low-quality firm’s reliance on mass advertising could be examined as equilibrium strategies, where neither firm can improve its perceived quality or profitability by deviating from its chosen strategy.

Moreover, the Nash equilibrium framework would provide deeper insights into the anchoring effect, where the low-quality firm adjusts its perceived quality to a fixed ratio (4/7) of the high-quality firm’s standard. By modeling this interaction as a non-cooperative game, we could explore how the anchoring effect emerges as an equilibrium outcome shaped by the firms’ strategic responses to each other’s advertising efforts. This would not only enhance the theoretical rigor of the study but also provide a more comprehensive understanding of the competitive dynamics in vertically differentiated markets.

In conclusion, incorporating the Nash equilibrium into the analysis would strengthen the study’s findings by offering a more precise and strategic perspective on the firms’ advertising decisions and their impact on perceived product quality. It would also highlight the interplay between competition, advertising strategies, and consumer perceptions, providing valuable insights for theoretical research and practical applications in marketing and competitive strategy.

### 3.3 Benchmark model

In solving the game, consider the demand faced by each firm. By the game rules, we believe the paramete r$s_{L}$ is firm *L*’s product quality, whereas the parameter$s_{H}$ is firm *H*’s quality with the condition that $0<s_{L}<s_{H}$. When the market is uncovered, some consumers do not purchase in this market. A consumer with an index $\theta_{L}$ purchasing the product with a requirement $\theta_{L}s_{L}-p_{L}=0$ will be indifferent between purchasing from firm L and not buying any. Therefore, any consumer with an index greater than $\theta_{L}$ will prefer to purchase from firm L rather than accept any. Analogously, a consumer with an index $\theta_{H}$ where $\theta_{H}s_{H}-p_{H}=\theta_{H}s_{L}-p_{L}$ will be indifferent between purchasing from firm H and firm L. Any consumer with an index higher than $\theta_{H}$ that will prefer to buy from firm H than from firm L. The parameter $q_{H}$ is defined as the set of all consumers with preferences greater than $\theta_{L}$. According to the condition mentioned above, firm profits in an uncovered market with the demand and cost condition are considered to be $\pi_{H}$ and $\pi_{L}$. Within game theory, both firms strongly desire to maximize profits in a duopolistic environment. The best reply function of the pricing game in the final stage is derived from the first-order conditions of each firm.

In this scenario, we assume that the two competitive firms produce distinct products with vertical differentiation. Theoretical and empirical considerations guide the choice of parameters. The vertical differentiation model accurately reflects market conditions where firms differentiate their products based on quality. The use of perceived quality as a key variable aligns with our research objective of understanding the impact of advertising on consumer perceptions. It is important to note that the product’s marginal production cost is considered zero. With these assumptions in mind, we can outline the following model setup.


$$\theta_{H}s_{H}-p_{H}=\theta_{H}s_{L}-p_{L}$$ (2)

$$\theta_{L}s_{L}-p_{L}=0$$ (3)

$$\underset{p_{H}}{\max}\pi_{H}=p_{H}q_{H}=p_{H}\left(1-\frac{p_{H}-p_{L}}{s_{H}-s_{L}}\right)$$ (4)

$$\underset{p_{L}}{\max}\pi_{L}=p_{L}q_{L}=p_{L}\left[\frac{p_{H}-p_{L}}{s_{H}-s_{L}}-\frac{p_{L}}{s_{L}}\right]$$ (5)

According to the Nash equilibrium, we are solving for the optimal prices with distinct qualities $p_{H}^{*}$ and $p_{L}^{*}$, as follows.


$$p_{H}^{*}=\frac{2(s_{H}-s_{L})s_{H}}{4s_{H}-s_{L}};p_{L}^{*}=\frac{(s_{H}-s_{L})s_{L}}{4s_{H}-s_{L}}$$

It is worth noting that both firms are faced with intense price competition. As a result, they are compelled to employ various strategies to enhance the quality of their products and differentiate themselves in the market. Consequently, the demand function can be expressed in terms of *S*_*H*_ and *S*_*L*_, representing the demand for high and low-quality firms, respectively. This result serves as a crucial mechanism for capturing the impact of product quality on consumer preferences and purchasing decisions.


$$q_{H}^{*}=\frac{2s_{H}}{4s_{H}-s_{L}};q_{L}^{*}=\frac{s_{H}}{4s_{H}-s_{L}}$$

The equilibrium profit function of both firms with high quality and low quality are:


$$\pi_{H}^{*}=\frac{4(s_{H}-s_{L})s_{H}^{2}}{{\left(4s_{H}-s_{L}\right)}^{2}};\pi_{L}^{*}=\frac{(s_{H}-s_{L})s_{H}s_{L}}{{\left(4s_{H}-s_{L}\right)}^{2}}$$

The first-order condition for the maximization of $\pi_{L}^{*}$ yields;


$$(s_{H}-2s_{L})(4s_{H}-s_{L})+2(s_{H}-s_{L})s_{L}=0$$ (6)

Note that equation (5) simplifies to the following equation $s_{L}=\frac{4}{7}s_{H}$. Besides, the first-order condition for maximization of $\pi_{H}^{*}$ is always higher than 0. Thus, in equilibrium, we have solved the following results $p_{H}^{*}=\frac{s}{4}$; $p_{L}^{*}=\frac{s}{14}$; $q_{H}=\frac{7}{12}$; $q_{L}=\frac{7}{24}$; $\pi_{H}^{*}=\frac{7s}{48}$; and $\pi_{L}^{*}=\frac{s}{48}$ (See Appendix 1, S1 Fig left and S1 Fig right in S1 File).

According to the above benchmark, it is easy to obtain that $\frac{\partial \pi_{H}^{*}}{\partial s_{H}}>0$, which means that firm H’s equilibrium profit function is a monotonic increasing function on the increase of quality H. However, firm L’s equilibrium profit function is a monotonic increasing function when $s_{L}\in (0,\frac{4s_{H}}{7}]$, whereas it shows a monotonic decreasing function when $s_{L}\in [\frac{4s_{H}}{7},s_{H}]$. It is considered that the firm demand is a function of consumer taste parameters. Firms can invest in persuasive advertising to influence consumer preferences favorably. For example, Firm H, which produces high-quality products, can utilize advertising strategies to attract more customers who are loyal to the high-quality brand by enriching their perception of taste $\theta_{H}$. Similarly, Firm *L*, specializing in low-quality products, can advertise to expand its customer base by targeting individuals whose taste preferences $\theta_{L}$ align with the low-quality brand. Through persuasive advertising, firms can also convert non-consumers into new customers who make purchases solely due to the influence of advertising. It implies that perceived product quality can be enhanced by applying persuasive advertising techniques. Considering these considerations, we can move on to the following proposition.

**Proposition 1:** *In a competition between a firm producing high-quality products and a rival firm producing low-quality products, the high-quality firm always possesses the impetus to enhance its perceived quality through persuasive advertising targeted at consumers. On the other hand, the low-quality rival firm will maintain its perceived quality at a fixed ratio of 4/7, anchoring it to the high-quality firm.*

Building upon Proposition 1 and Appendix 2 (See S2 Fig in S1 File), we can observe that when the low-quality firm (Firm L) quality falls below 4/7 compared to the high-quality firm, it is motivated to employ persuasive advertising techniques to amplify its perceived quality. This result highlights the anchoring effect, wherein the low-quality firm finds incentives to improve its perceived quality in response to its competitor’s higher quality. This dynamic suggests that persuasive advertising and the anchoring effect drive firms to continuously enhance their perceived quality, creating a competitive environment where both firms strive to improve their offerings.

### 3.4 The model with mass persuasive advertising

Mass persuasive advertising aims to reach a broad audience to create brand awareness and influence consumer perceptions. Based on the Hierarchy of Effects Model [37], we hypothesize that mass advertising can enhance perceived quality through repeated exposure and message reinforcement.

H1: *Firms employing mass persuasive advertising will experience increased perceived product quality due to enhanced brand awareness and message repetition.*

Our framework assumes that consumers perceive these commodities as goods, while non-consumers view them as undesirable. Within the market, there exists a pool of non-consumers whose preferences can be influenced and molded. It is within this context that firms utilize persuasive advertising strategies to convince non-consumers that what they previously deemed unfavorable is, in reality, beneficial. By employing persuasive advertising, firms can expand the overall market size by attracting new customers who were previously non-consumers. In our model, when consumers have non-unit demands, persuasive advertising can effectively increase demand by inducing new customers to enter the market. It is important to note that in this scenario, the perceived quality of a firm’s products changes as a result of the persuasive advertising efforts. Consequently, both firms can choose marketing strategies that include or exclude advertising, which influences the overall game dynamics between the two firms. The possible outcomes of both firms employing mass advertising or not can be represented in the game array presented in Table 1. The superscript M indicates mass advertising. The upper *S* means the increased perceived from *s* to *s* + *a*.

**Table 1:** The game array of both firm’s profit with or without mass advertising.

	Without advertising $s_{L}^{0}$	Mass advertising $S_{L}^{M}$
**Without advertising** $s_{H}^{0}$	**A** $\left(\pi_{H}^{0},\pi_{L}^{0}\right)$	**B** $\left(\pi_{H}^{0},\pi_{L}^{M}\right)$
**Mass advertising** $S_{H}^{M}$	**C** $\left(\pi_{H}^{M},\pi_{L}^{0}\right)$	**D** $\left(\pi_{H}^{M},\pi_{L}^{M}\right)$

From the array mentioned above, both firms could sell products to the consumers without mass advertising, or one could send mass advertising to all the consumers. Even the prisoner’s dilemma occurs when both firms send mass advertising to the consumers to improve the quality of their products.

To differentiate between firms’ qualities with and without persuasive advertising, we define the following $s_{i}(i=H,L)$ as the firm’s quality after using persuasive advertising. We will explore the fact that when firm L does not use persuasive advertising to increase its quality, even firm H has enough power to send persuasive advertising. We define the enhanced perceived quality of the product receiving the persuasive advertising considered to be $a_{H}$ or $a_{L}$. Likewise, any consumer with an index greater than $\theta_{L}$ will prefer to purchase from firm L than that do not buy at all. Analogously, a consumer with an index of $\theta_{H}^{M}$, where the equation $\theta_{H}^{M}(s_{H}+a_{H})-p_{H}=\theta_{H}^{M}s_{L}-p_{L}$ will be indifferent between purchasing from firm H and purchasing from firm L. Any consumer with an index higher than $\theta_{H}^{M}$ that will prefer to buy from firm H rather than firm L.


$$\theta_{H}^{M}(s_{H}+a_{H})-p_{H}^{M}=\theta_{H}^{M}s_{L}-p_{L}$$ (7)

$$\theta_{L}s_{L}-p_{L}=0$$ (8)

$$\underset{p_{H}}{\max}\pi_{H}=p_{H}^{M}q_{H}-\frac{k}{2}a_{H}^{2}-\lambda =p_{H}^{M}\left(1-\frac{p_{H}^{M}-p_{L}}{s_{H}+a_{H}-s_{L}}\right)-\frac{k}{2}a_{H}^{2}-\lambda$$ (9)

According to the first-order condition on price, it is easy to obtain the following expression (See Appendix 3 in S1 File).


$$\pi_{H}^{*}=\frac{4{\left(s_{H}+a_{H}\right)}^{2}(s_{H}+a_{H}-s_{L})}{{\left[4(s_{H}+a_{H})-s_{L}\right]}^{2}}-\frac{k}{2}a_{H}^{2}-\lambda$$

Likewise, it is easy to obtain that $\frac{\partial \pi H}{\partial a_{H}}|a_{H}=0>0$. This implies that a firm producing high-quality products is consistently motivated to enhance its perceived quality by implementing extensive persuasive advertising campaigns targeted at consumers. As a result, Decision C, which involves mass advertising, remains a superior choice for the firm compared to Decision A, as indicated in Table 1. This reaffirms the effectiveness of persuasive advertising in elevating the perceived quality of the firm’s products, thereby allowing it to maintain a competitive edge in the market.

**Proposition 2:** *In the scenario where a low-quality firm chooses not to employ mass advertising to enhance its perceived quality, the firm with high-quality will consistently have the impetus to utilize mass advertising to increase its perceived quality.*

According to proposition 2, when a firm opts not to invest in mass advertising despite having lower-quality products, it essentially relinquishes the opportunity to influence consumer perceptions and shape the market narrative. In contrast, a firm with high-quality offerings recognizes the strategic advantage of leveraging mass advertising to communicate the inherent value of its products and establish itself as a dominant player in the market. By consistently saturating the market with compelling advertising campaigns, the high-quality firm effectively reinforces its brand image and fosters positive associations with its products in the minds of consumers. Moreover, the absence of mass advertising by the low-quality firm inadvertently creates a power vacuum within the market, allowing the high-quality firm to capitalize on this gap by intensifying its advertising efforts. As consumers are bombarded with persuasive messages highlighting the superiority of the high-quality firm’s products, their perception of value and desirability naturally tilts in favor of the advertised offerings. Mass advertising is a powerful tool for a high-quality firm to solidify its competitive position and attract a larger share of the consumer base.

Additionally, the decision of the low-quality firm to refrain from mass advertising can be perceived as a strategic misstep, further amplifying the disparity in perceived quality between the two firms. Consumers tend to associate visibility and promotional activities with credibility and legitimacy, and the absence of such efforts by the low-quality firm may raise doubts about the reliability and trustworthiness of its products. In contrast, the high-quality firm’s relentless investment in mass advertising reinforces the perceived value of its offerings. It instills a sense of confidence and assurance among consumers, thereby consolidating its market leadership position.

Overall, the scenario described underscores the symbiotic relationship between mass advertising, perceived quality, and competitive dynamics within the market. While a firm’s decision to forego mass advertising may seem like a cost-saving measure in the short term, it inadvertently cedes ground to competitors and undermines its long-term viability. In contrast, firms that recognize the strategic importance of mass advertising in shaping consumer perceptions and driving demand are better positioned to maintain a competitive edge and thrive in the marketplace. An interesting condition to consider is when the firm selling high-quality products deliberately decides not to rely on persuasive advertising to improve its perceived quality. In such a case, examining whether the firm selling low-quality products still possesses the driving force to enhance its perceived quality becomes crucial. Expanding on Proposition 2, the competitive dynamics may shift when the high-quality firm abstains from using persuasive advertising tactics to improve its perceived quality. It raises the question of whether the low-quality firm has the innate motivation or capabilities to invest in strategies that can effectively increase its perceived quality without persuasive advertising. This exploration is essential in understanding how firms adapt their marketing strategies and compete within the market landscape.

Likewise, any consumer with an index greater than $\theta_{L}^{M}$ will prefer to purchase from firm L than that and not buy at all, that is $\theta_{L}^{M}(s_{L}+a_{L})-p_{L}=0$. Analogously, a consumer with an index $\theta_{H}$ and the equation $\theta_{H}s_{H}-p_{H}=\theta_{H}(s_{L}+a_{L})-p_{L}$ will be indifferent between purchasing from firm H and firm L when firm L sends persuasive mass advertising to consumers. Any consumer with an index higher than $\theta_{H}$ that will prefer to buy from firm H rather than firm L. Therefore, the model can be expressed as follows.


$$\theta_{H}-p_{H}=\theta_{H}(s_{L}+a_{L})-p_{L}^{M}$$ (10)

$$\theta_{L}^{M}(s_{L}+a_{L})-p_{L}^{M}=0$$ (11)

$$\underset{p_{L}}{\max}\pi_{L}=p_{L}^{M}q_{L}-\frac{k}{2}a_{L}^{2}-\lambda =p_{L}^{M}\left(\frac{p_{H}-p_{L}^{M}}{s_{H}-s_{L}-a_{L}}-\frac{p_{L}^{M}}{a_{L}}\right)-\frac{k}{2}a_{L}^{2}-\lambda$$ (12)

According to the first-order condition on the price of firm L’s product, it is easy to obtain the following expression (See Appendix 3 in S1 File).


$$\pi_{L}^{*}=\frac{s_{H}(s_{H}-s_{L}-a_{L})(s_{L}+a_{L})}{{\left[4s_{H}-(s_{L}+a_{L})\right]}^{2}}-\frac{k}{2}a_{L}^{2}-\lambda$$

Likewise, it is easy to obtain that $\frac{\partial \pi_{L}}{\partial a_{L}}|a_{L}=0=s_{H}(4s_{H}-7s_{L})$. That means as long as $4s_{H}-7s_{L}>0$ $\frac{\partial \pi_{L}}{\partial a_{L}}|a_{L}=0>0$. In this condition, the firm’s decision B is always better than decision A.

**Proposition 3:** *When the firm selling low-quality products has a market share of less than 4/7 compared to the high-quality firm, it will consistently be driven to enhance its perceived quality through mass persuasive advertising campaigns. However, note that a low-quality firm typically refrains from sending mass persuasive advertising to improve its perceived quality.*

Considering this proposition, we can infer that if the firm selling high-quality products demonstrates a strong driving force to improve its perceived quality, the low-quality firm will also feel compelled to send persuasive advertising to elevate its quality perception. This situation often leads to a prisoner’s dilemma, where both firms opt to send persuasive advertising to consumers. Consequently, modifying the existing model to accommodate the scenario where both firms engage in mass persuasive advertising efforts becomes necessary. Expanding on Proposition 3, when the market share of the low-quality firm falls below 4/7 in comparison to the high-quality firm, it faces intense competition and a potential loss of market share. As a result, the low-quality firm is motivated to invest in persuasive advertising to bridge the quality perception gap between itself and the high-quality firm. This dynamic further reinforces the complex interplay between marketing strategies, competition, and consumer perception within the market.


$$\theta_{H}^{M}(s_{H}+a_{H})-p_{H}^{M}=\theta_{H}^{M}(s_{L}+a_{L})-p_{L}^{M}$$ (13)

$$\theta_{L}^{M}(s_{L}+a_{L})-p_{L}^{M}=0$$ (14)

$$\underset{p_{H}^{M}}{\max}\pi_{H}^{M}=p_{H}^{M}q_{H}^{M}-\frac{k}{2}a_{H}^{2}-\lambda =p_{H}^{M}\left[1-\frac{p_{H}^{M}-p_{H}^{M}}{\left(s_{H}+a_{H})-(s_{L}+a_{L}\right)}\right]-\frac{k}{2}a_{H}^{2}-\lambda$$ (15)

$$\underset{p_{L}^{M}}{\max}\pi_{L}^{M}=p_{L}^{M}q_{L}^{M}-\frac{k}{2}a_{L}^{2}-\lambda =p_{L}^{M}\left[\frac{p_{H}^{M}-p_{L}^{M}}{\left(s_{H}+a_{H})-(s_{L}+a_{L}\right)}-\frac{p_{L}^{M}}{s_{L}+a_{L}}\right]-\frac{k}{2}a_{L}^{2}-\lambda$$ (16)

According to the first-order condition on price, it is easy to obtain the following expression (See Appendix 3 in S1 File).


$$\pi_{H}^{*}=\frac{4{\left(s_{H}+a_{H}\right)}^{2}(s_{H}+a_{H}-s_{L}-a_{L})}{{\left[4(s_{H}+a_{H})-(s_{L}+a_{L})\right]}^{2}}-\frac{k}{2}a_{H}^{2}-\lambda$$ (17)

$$\pi_{L}^{*}=\frac{\left(s_{H}+a_{H})(s_{H}+a_{H}-s_{L}-a_{L})(s_{L}+a_{L}\right)}{{\left[4(s_{H}+a_{H})-(s_{L}+a_{L})\right]}^{2}}-\frac{k}{2}a_{L}^{2}-\lambda$$ (18)

Additionally, consider the condition when $a_{L}=0$, from equation (18), only when

$\frac{\left[8(s_{H}+a_{H})(s_{H}+a_{H}-s_{L})+{\left(s_{H}+a_{H}\right)}^{2}\right]\left[4(s_{H}+a_{H})-s_{L}\right]-32{\left(s_{H}+a_{H}\right)}^{2}(s_{H}+a_{H}-s_{L})}{a_{H}{\left[4(s_{H}+a_{H})-s_{L}\right]}^{3}}>k$, we can obtain that $\frac{\partial \pi_{H}^{*}}{\partial a_{H}}|a_{H}=0>0$ (Appendix 3 in S1 File). That means only in this situation the firm’s decision can be changed from decision C to decision D. Likewise, consider the condition when $a_{H}=0$, from the equation (18), only when

That means the following expression $\frac{\partial \pi_{L}^{*}}{\partial a_{L}}|a_{H}=0>0$ yields the next term only when


$$\frac{s_{H}(s_{H}-2s_{L}-2a_{L})\left[4s_{H}-(s_{L}+a_{L})\right]+2s_{H}(s_{H}-s_{L}-a_{L})(s_{L}+a_{L})}{a_{L}{\left[4s_{H}-(s_{L}+a_{L})\right]}^{3}}>k>0$$

We can obtain that $\frac{\partial \pi_{L}^{*}}{\partial a_{L}}|a_{H}=0>0$ (Appendix 3). In this situation, only the firm’s decision can be changed from decision B to decision D.

**Proposition 4:** *The decisions of both firms are influenced by the propagation coefficient of mass persuasive advertising, highlighting the importance of technology improvement and minimizing the propagation coefficient. Furthermore, the transmission cost associated with advertising can significantly impact the equilibrium profit of the firm. In cases where the propagation coefficient is excessively high, both firms will cease their efforts to enhance their perceived qualities through persuasive advertising to consumers.*

Proposition 4 emphasizes the pivotal role played by the propagation coefficient of mass persuasive advertising in shaping the decisions of firms within a competitive market landscape. Expanding upon this proposition, it becomes evident that technological advancements and strategies to minimize the propagation coefficient are imperative for firms striving to maintain a competitive edge and maximize profitability. Firstly, the propagation coefficient represents the extent to which persuasive advertising messages disseminate and influence consumer perceptions across the market. In scenarios where this coefficient is high, advertising messages have a widespread and profound impact, significantly shaping consumer preferences and driving market dynamics. However, this widespread influence can also lead to heightened competition and escalating transmission costs, posing challenges for firms aiming to differentiate themselves and extract value from their advertising investments.

Technological innovation is crucial in mitigating the adverse effects of a high propagation coefficient. By developing more targeted and efficient advertising strategies, firms can minimize the dilution of their messaging and ensure that their efforts resonate with the intended audience segments. Advanced data analytics, machine learning algorithms, and personalized advertising platforms enable firms to tailor their messages to individual consumer preferences, thereby enhancing the effectiveness of their advertising campaigns while optimizing resource allocation. Furthermore, the transmission cost associated with advertising constitutes a significant determinant of firms’ equilibrium profits and strategic decisions. As the propagation coefficient increases, so does the intensity of competition for consumer attention and market share, leading to escalating transmission costs for firms engaged in mass persuasive advertising. In such circumstances, firms may be trapped in a costly arms race, where escalating advertising expenditures yield diminishing returns regarding perceived quality enhancement and market penetration. In cases where the propagation coefficient reaches excessively high levels, firms may reach a point of diminishing returns, where further investments in persuasive advertising fail to yield commensurate benefits in terms of perceived quality enhancement or competitive advantage. Recognizing the diminishing marginal utility of advertising expenditures, firms may reassess their strategic priorities and reallocate resources toward alternative avenues for value creation and differentiation.

Ultimately, Proposition 4 underscores the dynamic interplay between technological innovation, transmission costs, and the propagation coefficient of mass persuasive advertising in shaping firms’ strategic decisions and market outcomes. By leveraging technology to optimize advertising efficiency, minimize transmission costs, and adapt to changing market dynamics, firms can enhance their competitive resilience and maximize profitability in an increasingly complex and competitive business environment. One crucial aspect is how the product’s perceived quality impacts both firms’ profitability. This result can be assessed by analyzing the first-order condition governing the firm’s increased quality, as outlined in equations (17) and (18). Expanding on Proposition 4, the propagation coefficient measures the effectiveness of mass persuasive advertising in influencing consumer perception. It becomes imperative for firms to constantly improve their technology and communication strategies to reduce this coefficient. By doing so, firms can enhance their competitive advantage by effectively shaping consumer perception and boosting profitability. However, it is essential to note that there is a threshold beyond which a high propagation coefficient becomes detrimental to both firms. When the coefficient is excessively high, diminishing returns may occur, rendering further investments in persuasive advertising ineffective. At this stage, firms may need to explore alternative strategies to maintain their competitive edge and profitability.

As described by Zong and Guo [38], the anchoring effect influences how consumers assimilate new information based on pre-existing standards. In advertising, we propose that the initial quality perception serves as an anchor, influencing how subsequent advertising messages are interpreted.

H3: *The anchoring effect will moderate the impact of advertising on perceived product quality, with lower-quality firms experiencing a fixed adjustment ratio (e.g., 4/7) in perceived quality.*

Equations (17) and (18) provide valuable insights in determining the impact of quality on firms’ profits. By analyzing these first-order conditions, we can gain a deeper understanding of how improving the perceived quality of their products directly affects the bottom line of both firms, further informing their strategic decisions in the market.

Here, we consider that $S_{H}=s_{H}+a_{H}$ and $S_{L}=s_{L}+a_{L}$. That means


$$\frac{\partial \pi_{H}^{*}}{\partial a_{H}}=\frac{\partial \pi_{H}^{*}}{\partial S_{H}};\frac{\partial \pi_{L}^{*}}{\partial a_{L}}=\frac{\partial \pi_{L}^{*}}{\partial S_{L}}$$

Then, we can obtain the following expression $a_{H}^{*}>a_{L}^{*}$. In equilibrium, the enhanced quality for the firm selling high-quality products will always be higher than that of a low-quality firm (See appendix 4).


$$Otherwise\;\frac{p_{H}^{M}}{p_{L}^{M}}=\frac{2S_{H}}{S_{L}}>\frac{Q_{H}}{Q_{L}}=2$$

This equation indicates that the profitability of a high-quality firm is predominantly determined by its higher price point. In contrast, the low-quality firm’s profitability relies more heavily on its perceived quality. Consequently, the firm selling high-quality products will possess a more potent driving force to enhance its pricing strategy through continuous improvements in product quality. On the other hand, low-quality firms will exert more outstanding efforts to boost sales volume. The high-quality firm’s ability to command a premium price is directly linked to consumers’ perception of superior quality. By consistently improving the quality of their products, they can justify and maintain higher price points, contributing to their profitability. In contrast, low-quality firms may struggle to compete based on price alone. Instead, they focus on improving their sales performance by implementing persuasive marketing strategies highlighting other aspects, such as affordability or unique selling points. While their profit margins may be lower than those of a high-quality firm, their emphasis on sales volume allows them to remain competitive. Therefore, in terms of driving forces, the firm selling high-quality products focuses more on price improvement through quality enhancement. In contrast, low-quality firms exert energy toward driving sales growth through persuasive advertising and marketing techniques. Both approaches cater to different market segments and contribute to the overall dynamics of competition within the industry.

**Proposition 5:** *The firm selling a high-quality product consistently demonstrates an intention to employ mass persuasive advertising as a means to enhance its perceived quality. In contrast, the low-quality firm is significantly less inclined to engage in extensive mass persuasive advertising efforts. Additionally, the high-quality firm typically sets higher prices to sustain its premium position in the market, while the low-quality firm focuses on improving sales through alternative means.*

This proposition emphasizes the apparent disparity in advertising intentions between the firms selling high-quality and low-quality products. The high-quality firm recognizes the value of utilizing targeted advertising campaigns to communicate the superior attributes of its products to consumers effectively. This strategic approach aligns with its objective of enhancing the perceived quality and maintaining a competitive advantage. Conversely, the low-quality firm faces different challenges and strategic priorities. Given its inherent limitations in product quality, it tends to prioritize sales improvements by exploring alternative marketing strategies instead of relying heavily on mass persuasive advertising. This approach aims to compensate for the quality gap and attract consumers through other appealing aspects, such as competitive pricing or unique features. Considering Proposition 5, it becomes crucial for firms to adopt a comprehensive and adaptive advertising strategy. They should constantly test and optimize their advertising campaigns to identify the most suitable target audience and tailor the messaging to resonate with consumer preferences. This dynamic approach enables firms to strike a delicate balance between maximizing the conversion rate of their advertising efforts and achieving sufficient exposure in the market. By finding this equilibrium, firms can effectively leverage targeted and mass advertising methods to optimize their marketing impact and drive business growth.

According to the first condition outlined in equations (17) and (18), a specific solution is not readily apparent. Consequently, to examine the impact of persuasive advertising on a firm’s profit, we define particular parameters. This allows us to evaluate the effects in practical terms. As a result, the comprehensive explanation of the equation yields the following illustrative examples within the program.

When we set the parameters $s_{H}=2,s_{L}=1,k=1$, we can obtain the following results $a_{H}^{*}=0.2729,a_{L}^{*}=0.0192,\pi_{H}^{*}=0.3603-$ $\;\lambda ,\pi_{L}^{*}=0.0416-\lambda$.

When $s_{H}=2,s_{L}=1,k=2$, we can obtain the following results $a_{H}^{*}=0.1379,a_{L}^{*}=0.0080,\pi_{H}^{*}=0.3440-\lambda ,\pi_{L}^{*}=0.0482-\lambda$.

Likewise, when $s_{H}=2,s_{L}=1,k=5$, we can obtain the following results as $a_{H}^{*}=0.0556,a_{L}^{*}=0.0027,\pi_{H}^{*}=0.3337-$ $\;\lambda ,\pi_{L}^{*}=0.0416-\lambda$. Similarly, when the parameter is set as λ=0.01 and the specific value of the solution is shown in S1 Table in Appendix 4 in S1 File.

**Proposition 6:** *The increase in the production cost parameter of advertising can lead to a decline in the enhanced qualities of both firms, particularly impacting the firm with low-quality products at a faster rate. Similarly, a rise in the transmission cost parameter of advertising has a detrimental effect on the equilibrium profit of both firms. When the transmission cost parameter becomes excessively high, the firm’s equilibrium profit may be lower than in a scenario without advertising.*

This proposition highlights a crucial managerial insight regarding the dual nature of advertising costs and their impact on a firm’s profitability. The bidirectional roles arise from the inherent nature of advertising costs. A compelling incentive exists for firms producing low-quality products to improve their advertising production cost. This is particularly evident due to the 4/7 ratio between low-quality and high-quality firms, suggesting a need for low-quality firms to allocate more resources towards advertising. Conversely, firms that produce high-quality products should prioritize expanding transmission and communication channels to reach their target consumers effectively. In today’s increasingly competitive market landscape, the strength of marketing communication directly correlates with brand influence. It enables firms to rapidly enhance brand awareness and establish a favorable position in the market.

The implications of Proposition 6 emphasize the vital importance of striking a balance between production cost and transmission cost parameters in advertising. Managers should carefully evaluate and optimize these parameters based on the quality of the products they offer. By doing so, firms can allocate resources effectively, enhance marketing effectiveness, and drive brand awareness and market success.

## 4 The model with Internet-based targeted persuasive advertising

Targeted advertising leverages data analytics to deliver personalized messages to specific consumer segments. Theoretical frameworks such as the Persuasion Knowledge Model suggest that personalized advertising can influence consumer perceptions by addressing individual preferences and reducing skepticism [39].

H2: *Firms employing Internet-based targeted persuasive advertising will experience a more significant increase in perceived product quality compared to mass advertising due to message personalization and relevance.*

Likewise, we define that the firm’s perceived quality changes from $s_{i}$ to $S_{i}$ when the firm sends targeted persuasive advertising to consumers. Therefore, both firms could choose marketing strategies without advertising or targeted advertising. The game array of both firms using targeted advertising can be shown in Table 2. The superscript T indicates targeted advertising.

**Table 2:** The game array of both firm’s profit with or without mass advertising.

	Without advertising $s_{L}^{0}$	Targeted advertising $S_{L}^{T}$
**Without advertising** $s_{H}^{0}$	_**A’**_ $\left(\pi_{H}^{0},\pi_{L}^{0}\right)$	_**B’**_ $\left(\pi_{H}^{0},\pi_{L}^{T}\right)$
**Targeted advertising** $S_{H}^{M}$	_**C’**_ $\left(\pi_{H}^{T},\pi_{L}^{0}\right)$	_**D’**_ $\left(\pi_{H}^{T},\pi_{L}^{T}\right)$

From the scenarios above, it is apparent that both firms can sell their products to consumers without utilizing targeted advertising, or one of the firms may choose to employ targeted advertising and reach out to all consumers. In an intriguing turn, a situation resembling the classic prisoner’s dilemma arises when both firms use targeted advertising to enhance the perceived quality of their products.

Our study employs a vertical differentiation model to examine the effects of advertising on firms with distinct product qualities. Theoretical insights from the Vertical Differentiation Theory [40] and Salop’s Circular City Model [41] inform our understanding of how firms position themselves based on product quality and the strategic use of advertising to differentiate their offerings.

H4: *Higher-quality firms benefit more from Internet-based targeted advertising in a competitive duopoly. In contrast, lower-quality firms will rely more on mass advertising to enhance perceived quality and compete effectively.*

When firms employ persuasive targeted advertising, it opens avenues for high-value and low-value product sellers to reach their intended consumers effectively. Conversely, when using mass advertising, both firms may attempt to elevate the perception of their products’ quality to differentiate themselves in the market. This dynamic highlights the strategic choices available to firms in leveraging both targeted and mass advertising methodologies. Note that they would make the changes in the parameters $b_{i}(i=H,L)$, which are considered to be the improved quality of the firm, both high quality and low quality, after targeted advertising. A consumer with an index$\theta_{H}$, where the equation $\theta_{H}^{T}(s_{H}+b_{H})-p_{H}^{T}=\theta_{H}^{T}s_{L}-p_{H}^{T}$ will be indifferent between purchasing from firm H and from firm L when firm H sends persuasive, targeted advertising to consumers. Any consumer with an index higher than $\theta_{H}$ that will prefer to buy from firm H rather than firm L.


$$\theta_{H}^{T}(s_{H}+b_{H})-p_{H}^{T}=\theta_{H}^{T}s_{L}-p_{L}$$ (19)

$$\theta_{L}s_{L}-p_{L}=0$$ (20)

$$\underset{p_{H}^{T}}{\max}\pi_{H}^{T}=p_{H}^{T}q_{H}^{T}=(p_{H}^{T}-\lambda )\left[1-\frac{p_{H}^{T}-p_{L}^{T}}{(s_{H}+b_{H})-s_{L}}\right]-\frac{k}{2}b_{H}^{2}$$ (21)

According to the first-order conditions on price, it is easy to obtain the following expression (See Appendix 5 in S1 File).


$$\pi_{H}^{T}=\frac{(s_{H}+b_{H}-s_{L}){\left[2(s_{H}+b_{H})-\lambda \right]}^{2}}{{\left[4(s_{H}+b_{H})-s_{L}\right]}^{2}}-\frac{k}{2}b_{H}^{2}$$ (22)

Likewise, when the condition $2s_{H}-\lambda >0$ is satisfied, the following expression $\frac{\partial \pi_{H}}{\partial b_{H}}|b_{H}=0>0$ is established (See Appendix 5 in S1 File). This means that a firm with high-quality always has a driving force to increase its perceived quality by sending Internet-based targeted persuasive advertising to consumers as long as the transmission cost parameter is not relatively high. The decision C’ is always better than that decision A’ (Table 2).

Consider the condition when firm L uses persuasive targeted advertising, whereas firm H keeps it unchanged. That means:


$$\theta_{H}^{T}s_{H}-p_{H}^{T}=\theta_{H}^{T}(s_{L}+b_{L})-p_{L}^{T}$$ (23)

$$\theta_{L}^{T}(s_{L}+b_{L})-p_{L}^{T}=0$$ (24)

$$\underset{p_{L}^{T}}{\max}\pi_{L}^{T}=p_{L}^{T}q_{L}^{T}=(p_{L}^{T}-\lambda )\left[\frac{p_{H}^{T}-p_{L}^{T}}{s_{H}-(s_{L}+b_{L})}-\frac{p_{L}^{T}}{s_{L}+b_{L}}\right]-\frac{k}{2}b_{L}^{2}$$ (25)

According to the first-order condition on price, it is easy to obtain the following expression (See Appendix 6 in S1 File).


$$\pi_{L}^{T}=\frac{s_{H}\left[s_{H}-(s_{L}+b_{L})\right]{\left[(s_{L}+b_{L})-2\lambda \right]}^{2}}{{\left[4s_{H}-(s_{L}+b_{L})\right]}^{2}(s_{L}+b_{L})}-\frac{k}{2}b_{L}^{2}$$ (26)

Likewise, as long as the following $s_{H}>s_{L}>2\lambda$ is satisfied, it is easy to obtain that the following expression $\frac{\partial \pi_{L}^{T}}{\partial b_{L}}|b_{L}=0>0$ holds. (See Appendix 6 in S1 File). This means that a firm with high-quality always has a driving force to increase its perceived quality by sending Internet-based targeted persuasive advertising to consumers as long as the transmission cost parameter is not relatively high. Decision C’ is always better than that decision A’.

While using persuasive targeted advertising, firms with high-value and low-value products can send their targeted advertising to the targeted consumer. The firm sends mass advertising to consumers with distinct attributes to differentiate the increased perceived quality. After targeted advertising, the parameter $b_{i}(i=H,L)$ considers the improved quality of the firm, both high quality and low quality. Likewise, any consumer with an index higher than $\theta_{L}^{T}$ will prefer to purchase from firm L than that do not buy at all, that is $\theta_{L}^{T}(s_{L}+b_{L})-p_{L}^{T}=0$. Analogously, the following equation $\theta_{H}^{T}(s_{H}+b_{H})-p_{H}^{T}=\theta_{H}^{T}(s_{L}+b_{L})-p_{H}^{T}$ for a consumer with an index $\theta_{H}$ will be indifferent between purchasing from firm H and firm L when firm L sends persuasive mass advertising to consumers. Any consumer with an index higher than $\theta_{H}$ that will prefer to buy from firm H rather than firm L. Therefore, the model can be expressed as follows.


$$\theta_{H}^{T}(s_{H}+b_{H})-p_{H}^{T}=\theta_{H}^{T}(s_{L}+b_{L})-p_{L}^{T}$$ (27)

$$\theta_{L}^{T}(s_{L}+b_{L})-p_{L}^{T}=0$$ (28)

$$\underset{p_{H}^{T}}{\max}\pi_{H}^{T}=p_{H}^{T}q_{H}^{T}=(p_{H}^{T}-\lambda )\left[1-\frac{p_{H}^{T}-p_{L}^{T}}{\left(s_{H}+b_{H})-(s_{L}+b_{L}\right)}\right]-\frac{k}{2}b_{H}^{2}$$ (29)

According to the first-order condition on price, it is easy to obtain the following expression (See appendix 7).


$$\underset{p_{L}^{T}}{\max}\pi_{L}^{T}=p_{L}^{T}q_{L}^{T}=(p_{L}^{T}-\lambda )\left[\frac{p_{H}^{T}-p_{L}^{T}}{\left(s_{H}+b_{H})-(s_{L}+b_{L}\right)}-\frac{p_{L}^{T}}{s_{L}+b_{L}}\right]-\frac{k}{2}b_{L}^{2}$$ (30)

According to the first-order condition on price, it is easy to obtain the following expression (See Appendix 7 in S1 File).


$$\pi_{H}^{T}=\frac{(s_{H}+b_{H}-s_{L}-b_{L}){\left[2(s_{H}+b_{H})-\lambda \right]}^{2}}{{\left[4(s_{H}+b_{H})-(s_{L}+b_{L})\right]}^{2}}-\frac{k}{2}b_{H}^{2}$$ (31)

$$\pi_{L}^{T}=\frac{(s_{H}+b_{H})\left[\left(s_{H}+b_{H})-(s_{L}+b_{L}\right)\right]{\left[(s_{L}+b_{L})-2\lambda \right]}^{2}}{{\left[4(s_{H}+b_{H})-(s_{L}+b_{L})\right]}^{2}(s_{L}+b_{L})}-\frac{k}{2}b_{L}^{2}$$ (32)

Likewise, as long as the condition $s_{H}>s_{L}>2\lambda$ is satisfied, it is easy to obtain that $\frac{\partial \pi_{L}^{T}}{\partial b_{L}}|b_{L}=0>0$ (See Appendix 7 in S1 File). This means that a firm with high-quality always has a driving force to increase its perceived quality by sending Internet-based targeted persuasive advertising to distinct consumers as long as the transmission cost parameter is not relatively high. The decision C’ is always better than that of A’.

The first-order condition on the increased quality reveals how quality affects both firms’ profits. The following expression can be obtained. Here, we consider that

$S_{H}^{T}=s_{H}+b_{H}$ $S_{L}^{T}=s_{L}+b_{L}$. That means the following equations are satisfied.


$$\frac{\partial \pi_{H}^{*}}{\partial b_{H}}=\frac{\partial \pi_{H}^{*}}{\partial S_{H}^{T}};$$ (33)

$$\frac{\partial \pi_{L}^{*}}{\partial b_{L}}=\frac{\partial \pi_{L}^{*}}{\partial S_{L}^{T}}$$ (34)

$$\frac{4S_{H}^{T}(4S_{H}^{T2}-3S_{H}^{T}S_{L}^{T}+2S_{L}^{T2})}{{\left(4S_{H}^{T}-S_{L}^{T}\right)}^{3}}-\frac{8S_{H}^{T}S_{L}^{T}\lambda +4\lambda S_{L}^{T2}+\lambda^{2}(4S_{H}^{T}-7S_{L}^{T})}{{\left(4S_{H}^{T}-S_{L}^{T}\right)}^{3}}=kb_{H}$$ (35)

$$\frac{S_{H}^{T2}(4S_{H}^{T}-7S_{L}^{T})}{{\left(4S_{H}^{T}-S_{L}^{T}\right)}^{3}}+\frac{4\lambda (S_{L}^{T}-\lambda )(4S_{H}^{T2}-3S_{H}^{T}S_{L}^{T}+2S_{L}^{T2})}{{\left(4S_{H}^{T}-S_{L}^{T}\right)}^{3}S_{L}^{T2}}=kb_{L}$$ (36)

Likewise, there are no analytic solutions to these equations. Therefore, we define some parameters to check the effect of persuasive advertising on the firm’s profit. Thus, we solve the equations with the “*fsolve function*” of Matlab 2103.

Only k=1,λ=0.3, k=2,λ=0.3, k=3,λ=0.3, there are serials of value solution, for instance, when k=1,λ=0.3, $S_{H}^{T}=2,S_{L}^{T}=1,aH=0.2611,aL=0.0369$; $S_{H}^{T}=2.2,S_{L}^{T}=1.2,aH=0.2661,aL=0.0304$ …; When k=2,λ=0.3 $S_{H}^{T}=2,S_{L}^{T}=1,aH=0.1310,aL=0.0153$; $S_{H}^{T}=2.2,S_{L}^{T}=1.2,aH=0.1338,aL=0.0121$ …; When k=3,λ=0.3, $S_{H}^{T}=2,S_{L}^{T}=1,aH=0.0525,aL=0.0051$; (See Appendix 8 in S1 File)

We can compare the results using mass advertising from the number of results. Obviously, with Internet-based targeted persuasive advertising, firms’ profits were reduced.


$$\pi_{H}^{T*}=0.3410>0.3603-\lambda =0.2603=\pi_{H}^{M*},\pi_{L}^{T*}=0.0286>0.0445-\lambda =-0.0545=\pi_{L}^{M*}$$

Both firms could reduce the waste of transmission costs and enhance their quality. Therefore, we can quickly obtain the following proposition 7.

**Proposition 7:** *When comparing mass persuasive advertising with internet-based targeted persuasive advertising, there is a notable reduction in the equilibrium increased intensity of high-quality firms, while the low-quality firm may see an improvement. Additionally, the equilibrium profit for both firms decreases. This finding validates the superiority of internet-based targeted persuasive advertising, primarily due to the reduced transmission cost associated with this approach.*

Proposition 7 delves into the comparative dynamics between traditional mass persuasive advertising and the more targeted approach of internet-based persuasive advertising within a competitive market setting. Expanding upon this proposition reveals the nuanced implications of each advertising strategy on firm behavior, market equilibrium, and overall profitability. Firstly, the transition from mass persuasive advertising to internet-based targeted advertising represents a shift towards more precise and efficient methods of reaching consumers. Unlike traditional mass advertising, which broadcasts messages to broad audience segments, internet-based targeted advertising leverages sophisticated algorithms and data analytics to tailor messages to individual consumer preferences and behaviors. This precision enhances the relevance and effectiveness of advertising efforts and reduces the likelihood of message dilution and wastage. Regarding market dynamics, Proposition 7 highlights a notable reduction in the equilibrium increased intensity of high-quality firms when transitioning to internet-based targeted advertising. This phenomenon can be attributed to several factors. Firstly, targeted advertising allows high-quality firms to reach their desired audience segments more effectively, thereby reducing the need for excessive advertising expenditures to gain visibility and market share.

Additionally, the ability to tailor messages to specific consumer preferences enhances the perceived value proposition of high-quality products or services, reducing the need for aggressive advertising tactics to differentiate themselves from competitors. Conversely, low-quality firms may experience an improvement in their equilibrium intensity under internet-based targeted advertising. This improvement stems from the ability of targeted advertising to identify and engage niche consumer segments that may be more receptive to the value proposition offered by low-quality products or services. By focusing their advertising efforts on these niche markets, low-quality firms can achieve a more efficient allocation of resources and potentially improve their market position despite inherent quality limitations.

However, despite the differential impacts on firm intensity, Proposition 7 also highlights a universal decrease in equilibrium profit for both high-quality and low-quality firms when transitioning to internet-based targeted advertising. This reduction in profitability can be attributed to the inherent trade-offs associated with targeted advertising. While targeted advertising offers greater precision and efficiency in reaching consumers, it also requires data acquisition, analytics, and technology infrastructure investments, which may offset the cost savings achieved through reduced transmission costs. Nevertheless, the overall finding of Proposition 7 validates the superiority of internet-based targeted persuasive advertising over traditional mass advertising, primarily due to the reduced transmission costs associated with this approach. By leveraging technology to deliver personalized and relevant consumer messages, firms can optimize their advertising investments, enhance consumer engagement, and improve market outcomes.

In conclusion, Proposition 7 underscores the transformative impact of internet-based targeted advertising on firm behavior and market dynamics. While the shift towards targeted advertising may entail specific challenges and trade-offs, its ability to improve advertising efficiency, enhance consumer engagement, and reduce transmission costs reaffirms its position as a superior advertising strategy in the contemporary business landscape.

In this context, specific consumers strongly favor higher price-to-quality ratios, making it easier to recognize and cater to their preferences through targeted advertising. The low transmission cost and precision targeting capabilities of internet-based targeted advertising facilitates the efficient delivery of quality-related information. Proposition 7 elucidates the advantages of internet-based targeted advertising compared to its mass advertising counterpart. Focusing on a specific group of consumers, this approach capitalizes on the 4/7 ratio, allowing the low-quality firm to successfully identify and engage with consumers who prioritize higher-quality products at more affordable prices. In this scenario, quality becomes a valuable targeting label, surpassing other labels such as age or personal interests, as it results in more accurate segmentation for consumers who are particularly sensitive to the quality-to-price ratio. Moreover, the precision targeting provided by this approach ensures a correct match between products and consumers seeking moderate-quality products or those who desire quality that surpasses their expectations. This capability to align product offerings with consumer needs is a distinctive advantage of targeted advertising, further solidifying its efficacy in driving consumer satisfaction and enhancing profitability for both high-quality and low-quality firms.

## 5 _Model extension_

The original assumption is that the advertising transmission cost $A_{y}=\lambda q$ is overly simplistic. According to the literature, non-linear changes should be considered, and the marginal return diminishing and threshold effects should be introduced. When $q\leq q_{threshold}$, we set $A_{y}=\lambda_{1}q-\beta_{1}q^{2}$; when$q>q_{threshold}$, we set $A_{y}=\lambda_{2}q+\beta_{2}(q-q_{threshold}{)}^{2}$. Here, $\lambda_{1}$ and $\lambda_{2}$ are the transmission cost coefficients in different stages, and the parameters $\beta_{1}$, $\beta_{2}$ are used to control the degree of marginal return changes, with $\beta_{1}>0$ and $\beta_{2}>0$. This modification can more accurately describe advertising cost changes with the number of consumers, reflecting different cost trends in the unsaturated and saturated market stages.

After considering the new advertising transmission cost and demand functions, when $q_{H}\leq q_{threshold}$, the profit function of Firm H and Firm L is


$$\pi_{H}=p_{H}q_{H}-\frac{k}{2}a_{H}^{2}-(\lambda_{1}q_{H}-\beta_{1}q_{H}^{2})$$ (37)

$$\pi_{L}=p_{L}q_{L}-\frac{k}{2}a_{L}^{2}-(\lambda_{1}q_{L}-\beta_{1}q_{L}^{2})$$ (38)

$$q_{H}=1-\frac{p_{H}-p_{L}}{\left(s_{H}+a_{H})-(s_{L}+a_{L}\right)}$$ (39)

$$q_{L}=\frac{p_{H}-p_{L}}{\left(s_{H}+a_{H})-(s_{L}+a_{L}\right)}-\frac{p_{L}}{s_{L}+a_{L}}$$ (40)

According to the first-order condition,


$$\{\begin{array}{c} {}*20c\frac{\partial \pi_{H}}{\partial p_{H}}=0\\ \frac{\partial \pi_{H}}{\partial a_{H}}=0\\ \frac{\partial \pi_{L}}{\partial p_{L}}=0\\ \frac{\partial \pi_{L}}{\partial a_{L}}=0 \end{array}$$ (41)

According to the above formula, the optimal price and quality improvement for firms H and L can be obtained. Therefore, during different stages of product promotion, the market saturation level and consumers’ responses to advertising vary, leading to other roles for targeted and mass advertising. Based on the text mentioned above, the differences in their roles during different periods are as follows:

### 5.1 In the initial stage of product promotion $\left(q\leq q_{threshold}\right)$

At this stage, the market is not yet saturated, and consumer demand remains to be tapped. Targeted advertising uses data analysis to precisely locate potential consumer groups, enabling it to reach people likely to be interested in the product efficiently. Taking a cosmetics brand as an example, social media platforms can push advertisements precisely according to users’ age, skin type, purchasing preferences, etc. For instance, it can push advertisements for oil-control and acne-treatment cosmetics to young consumers with oily skin. This precise push makes each advertisement more effective in attracting target consumers, and the advertising transmission cost is relatively low. Due to the accurate positioning, the sales volume increases significantly with each additional consumer, resulting in increasing marginal returns.

Meanwhile, it helps the brand quickly build awareness among the target customer group and cultivate early-stage user loyalty. Mass advertising can also play a particular role during this stage. By spreading widely, it can enhance overall brand awareness and let more consumers know about the product’s existence. For example, large-scale promotion on social media can rapidly expand the brand’s influence and attract many new consumers to purchase the product. However, compared with targeted advertising, mass advertising has weaker targeting. It may deliver advertisements to many consumers with little interest in the product, leading to some waste of advertising resources. Nevertheless, in the initial stage of market development, its broad coverage can accumulate a specific user base for the brand, laying a foundation for subsequent development.

### 5.2 In the stage of gradual market saturation $\left(q>q_{threshold}\right)$

As the market gradually saturates, consumers become less sensitive to advertising, and the effectiveness of mass advertising is significantly reduced. At this time, the advantages of targeted advertising become more prominent. It can deeply explore niche markets and push highly personalized advertising content to specific consumer groups. For example, a cosmetics brand can push advertisements for high-end anti-aging series to mature women who pursue high-quality products and have anti-aging needs. By precisely targeting these consumers with specific product demands, targeted advertising can find its target customers in a highly competitive market, improving the conversion rate of advertisements. Despite the saturated market, targeted advertising can still maintain a specific sales growth to some extent due to its precision, delaying the speed of diminishing marginal returns. It enhances the brand’s competitiveness in niche markets more effectively than mass advertising. In the saturated market stage, mass advertising faces many challenges. To attract more consumers, brands need to increase advertising channels, such as placing advertisements on TV, magazines, and other media, and they also need to create more attractive advertising content, which significantly increases the advertising transmission cost. However, due to the near-saturation of the market, even with a significant investment in advertising expenses, the sales growth becomes slow, and the marginal returns of advertising decrease significantly. For example, after a cosmetics brand conducts large-scale advertising on multiple media platforms, although the number of consumers reached by the advertisements further increases, the revenue growth brought by new consumers cannot offset the increase in advertising costs, resulting in slow or even negative profit growth. Mass advertising has difficulty reaching target customers accurately in this stage, which is likely to cause a waste of resources, and its contribution to brand profits gradually decreases.

In the initial stage of product promotion, targeted and mass advertising contribute to product promotion. However, targeted advertising performs better in increasing marginal returns with its precise positioning and lower transmission costs. In the saturated market stage, targeted advertising can better adapt to market changes and maintain specific sales growth through precise pushing. In contrast, mass advertising faces the dilemma of rising costs and diminishing returns, and its role is relatively weakened.

## 6 _Conclusion_

The vertical differential market sheds light on the intricate dynamics of competitive duopolistic firms employing persuasive advertising alongside vertical product differentiation. Without advertising, a firm’s quality level is pivotal, with the high-quality firm consistently maintaining superiority. At the same time, the low-quality counterpart anchors its perceived quality at 4/7 of the high-quality firm’s standard, known as the anchoring effect. Upon integrating persuasive advertising, both firms endeavor to elevate their perceived quality. Yet, when the transmission cost surpasses advertising’s production cost, the high-quality firm might opt out of mass advertising. At the same time, low-quality advertising may intensify persuasive advertising efforts, especially if it falls below the 4/7 quality threshold. However, precise quality control is challenging, and targeted advertising proves beneficial in aligning products with consumers’ price preferences and specific quality perceptions. Targeted advertising is a potent tool in influencing perceived quality, a strategic maneuver in the competitive landscape. By employing targeted persuasive advertising, firms don’t alter actual product quality; instead, they shape consumer perception, effectively altering market demand. Conversely, targeted quality pertains to actual product improvements. For low-quality firms, establishing an accurate anchor through targeted advertising is critical for enhancing perceived quality and meeting consumer preferences.

The study’s findings reveal several key insights into the relationship between persuasive advertising, vertical differentiation, and perceived product quality. First, mass and Internet-based targeted persuasive advertising can enhance perceived quality, but their effectiveness varies depending on the firm’s product quality and advertising strategy. For the high-quality firm, targeted advertising proves to be more advantageous, as it allows the firm to reach specific consumer segments that highly value the price-to-quality ratio. This strategy enhances perceived quality, reduces advertising costs, and improves profitability. In contrast, the low-quality firm tends to rely more on mass advertising, mainly when its product quality falls below the 4/7 threshold. This reliance on mass advertising is driven by the need to compensate for the lower actual quality of the product and establish a competitive position in the market. The study also highlights the anchoring effect, where the low-quality firm consistently adjusts its perceived quality to 4/7 of the high-quality firm’s standard. This effect has significant implications for competitive dynamics, shaping how firms position their products and allocate resources to advertising. The findings suggest that low-quality firms can benefit from targeted advertising by establishing an accurate anchor for perceived quality and meeting consumer preferences. Furthermore, the study underscores the strategic importance of selecting the appropriate advertising approach to maximize perceived product quality and gain a competitive advantage. Internet-based targeted advertising, in particular, offers several benefits, including improved brand loyalty, enhanced perceived value, and a narrower target market. However, the study also acknowledges potential drawbacks, such as privacy concerns and product quality issues, which must be addressed to realize the full benefits of targeted advertising.

This model offers an alternative view of the nexus between advertising and product quality. Leveraging Internet-based targeted advertising can reduce costs and enhance profits for both firms, with low-quality producers potentially benefitting more, making them inclined towards adopting such strategies. Conversely, high-quality producers may prefer mass advertising. Internet-based targeted advertising brings advantages like improved brand loyalty, perceived value, and a narrower target market. As AI progresses online, markets become more concentrated, yet potential drawbacks like privacy concerns and product quality issues must be addressed. Future studies should delve into these aspects for a comprehensive understanding, aiding in informed decision-making regarding AI-targeted advertising strategies.

This study investigates the differential impacts of mass persuasive advertising and Internet-based targeted persuasive advertising on firms with distinct product qualities within a competitive duopoly, focusing mainly on the anchoring effect on perceived quality. Our findings contribute to the theoretical understanding of advertising strategies, provide practical guidance for firms, and outline directions for future research. There are some significant research studies in this paper. Firstly, this study contributes to the Hierarchy-of-Effects Model and the Elaboration Likelihood Model [42] by demonstrating how different advertising strategies influence perceived product quality through cognitive and affective pathways. Our results indicate that Internet-based targeted advertising, due to its personalized and relevant messages, is more effective in enhancing perceived quality than mass advertising. Secondly, our results extend the application of perceived value [43] to advertising strategies, providing empirical evidence that initial quality perceptions act as anchors, influencing how subsequent advertising messages are interpreted. This adds a new dimension to understanding consumer judgment and decision-making in marketing contexts. Thirdly, the study adds to the Vertical Differentiation Theory by highlighting the strategic use of advertising to differentiate product qualities in a competitive market. Our findings show that higher-quality firms benefit more from targeted advertising, while lower-quality firms rely on mass advertising to enhance perceived quality and compete effectively.

## 7 _Research limitations and future directions_

Unique consumer preferences, personal values, and lifestyle choices significantly influence how advertising is perceived and its effectiveness. However, the extent to which these idiosyncratic attributes impact advertising outcomes remains underexplored, particularly in vertical markets with distinct product qualities. Future research should investigate how data-driven personalization efforts can align advertising strategies with individual consumer preferences to enhance perceived quality. Secondly, consumer ideologies, including deeply held beliefs, values, and cultural norms, significantly influence purchasing decisions. Future studies should explore how firms can leverage consumer ideologies, such as ethical sourcing or sustainability, in their advertising strategies to foster brand loyalty and resonate with value-driven consumers. Thirdly, as consumer preferences vary across different cultural contexts, future research should examine how these differences affect advertising design and effectiveness. Insights into these variations will be valuable for multinational companies looking to optimize their advertising strategies in diverse markets. Finally, the growing prevalence of AI-driven targeted advertising raises concerns about consumer privacy and autonomy. Future research should address the ethical implications of personalized advertising practices, explore the balance between personalization and privacy, and propose regulatory measures to ensure responsible advertising practices.

In conclusion, integrating idiosyncratic attributes and consumer ideologies into future research on persuasive advertising strategies will significantly enhance the depth and breadth of the analysis. These factors are crucial in shaping consumer perceptions and advertising responses, providing theoretical insights and practical guidance for firms. Additionally, as AI-driven targeted advertising evolves, understanding the interaction between consumer preferences, values, and regulatory frameworks will be essential for developing effective and responsible advertising strategies. The evolving landscape of AI-driven targeting and ethical considerations presents additional avenues for future research [44]. As firms continue to innovate in their advertising strategies, understanding the interaction between consumer preferences, values, and regulatory frameworks will be critical to developing effective and responsible advertising practices. Future research should explore these dynamics to inform the ongoing innovation in advertising practices, ensuring alignment with ethical standards and consumer expectations.

## Supporting information

S1 File **Appendix 1**. Proof of equilibrium firm’s profit. **S1 Fig**. 1 (left) Firm H’s profit changes with sH and sL. **S1 Fig**. 1 (right) Firm L’s profit changes with sH and sl. **Appendix 2**. Program of firm L changes with sH and sL. **S2 Fig**. 2 Contour map of firm L changes with sH and sL. **Appendix 3**. First order condition of firm H and firm L. **Appendix 4**. Proof of firm’s profit changes with variable parameters. **S1 Table**. The firm’s profit changes with variable parameters **Appendix 5**. Proof of firm H’s quality changes in specific condition. **Appendix 6**. Program for value solution equations. **Appendix 7**. First order condition of both firm’s changed quality. **Appendix 8**. Program for the equation to solve both firms’ profit.
(DOCX)
