# Supplementary material for: Differential role of internet-based targeted persuasive advertising versus mass advertising on firms with unique qualities in an anchoring perspective
Source: PLoS One. 2025 Jul 1;20(7):e0325552. doi: 10.1371/journal.pone.0325552 (PMC12212545; doi:10.1371/journal.pone.0325552)
Supplement: S1 File — Appendix 1. Proof of equilibrium firm’s profit. S1 Fig. 1 (left) Firm H’s profit changes with sH and sL. S1 Fig. 1 (right) Firm L’s profit changes with sH and sl. Appendix 2. Program of firm L changes with sH and sL. S2 Fig. 2 Contour map of firm L changes with sH and sL. Appendix 3. First order condition of firm H and firm L. Appendix 4. Proof of firm’s profit changes with variable parameters. S1 Table. The firm’s profit changes with variable parameters Appendix 5. Proof of firm H’s quality changes in specific condition. Appendix 6. Program for value solution equations. Appendix 7. First order condition of both firm’s changed quality. Appendix 8. Program for the equation to solve both firms’ profit. (DOCX) [file pone.0325552.s001.docx]

**Supporting information:**

**Appendix 1.**

Both firms can be expressed in the following expression.

$$\max_{p_{H}} \pi_{H}=p_{H}q_{H}=p_{H}\left(1-\frac{p_{H}-p_{L}}{s_{H}-s_{L}}\right)$$

$$\max_{p_{L}} \pi_{H}=p_{L}q_{L}=p_{L}\left(\frac{p_{H}-p_{L}}{s_{H}-s_{L}}-\frac{p_{L}}{s_{L}}\right)$$

According to the first-order condition on the price,

$$\begin{matrix} \frac{\partial\pi_{H}}{\partial p_{H}}=\left(1-\frac{p_{H}-p_{L}}{s_{H}-s_{L}}\right)-\frac{p_{H}}{s_{H}-s_{L}}=0; \\ \frac{\partial\pi_{L}}{\partial p_{L}}=\left(\frac{p_{H}-p_{L}}{s_{H}-s_{L}}-\frac{p_{L}}{s_{L}}\right)-p_{L}\left(\frac{1}{s_{H}-s_{L}}+\frac{1}{s_{L}}\right)=0 \end{matrix}$$

That means

$p_{H}^{*}=\frac{2(s_{H}-s_{L})s_{H}}{4s_{H}-s_{L}}$;$p_{L}^{*}=\frac{(s_{H}-s_{L})s_{L}}{4s_{H}-s_{L}}$

Therefore, both firms’ equilibrium sales can be expressed as follows.

$q_{H}=1-\frac{p_{H}-p_{L}}{s_{H}-s_{L}}=1-\frac{\frac{2(s_{H}-s_{L})s_{H}}{4s_{H}-s_{L}}-\frac{(s_{H}-s_{L})s_{L}}{4s_{H}-s_{L}}}{s_{H}-s_{L}}=\frac{2s_{H}}{4s_{H}-s_{L}}$;

$$q_{L}=\frac{p_{H}-p_{L}}{s_{H}-s_{L}}-\frac{p_{L}}{s_{L}}=\frac{2s_{H}-s_{L}}{4s_{H}-s_{L}}-\frac{(s_{H}-s_{L})}{4s_{H}-s_{L}}=\frac{s_{H}}{4s_{H}-s_{L}}$$

Both firm’s equilibrium profit can be expressed as follows.

$\pi_{H}^{*}=\frac{4(s_{H}-s_{L})s_{H}^{2}}{(4s_{H}-s_{L})^{2}}$;$\pi_{L}^{*}=\frac{(s_{H}-s_{L})s_{H}s_{L}}{(4s_{H}-s_{L})^{2}}$

$$\frac{\partial\pi_{H}^{*}}{\partial s_{H}}=4\frac{\left[(s_{H}-s_{L})s_{H}^{2}\right]'(4s_{H}-s_{L})^{2}-(s_{H}-s_{L})s_{H}^{2}\left[(4s_{H}-s_{L})^{2}\right]'}{(4s_{H}-s_{L})^{4}}=4\frac{s_{H}^{2}(4s_{H}-3s_{L})+2s_{H}s_{L}^{2}}{(4s_{H}-s_{L})^{3}}$$

$$\frac{\partial\pi_{L}^{*}}{\partial s_{L}}=\frac{\left[(s_{H}-s_{L})s_{H}s_{L}\right]'\left[(4s_{H}-s_{L})^{2}\right]-\left[(s_{H}-s_{L})s_{H}s_{L}\right]\left[(4s_{H}-s_{L})^{2}\right]'}{(4s_{H}-s_{L})^{4}}=0$$

Therefore,$p_{H}^{*}=\frac{s}{4};p_{L}^{*}=\frac{s}{14}$;$q_{H}=\frac{7}{12}$;$q_{L}=\frac{7}{24}$

$$\pi_{H}^{*}=\frac{7s}{48};\pi_{L}^{*}=\frac{s}{48}$$

To show the relationship between $\pi_{H}^{*}$ and$\pi_{L}^{*}$, the following figures (S Fig. 1 left and S Fig. 1 right) reveal both firms’ profits changing with sH and sL.


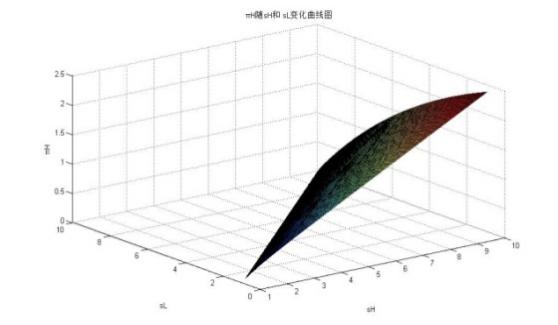

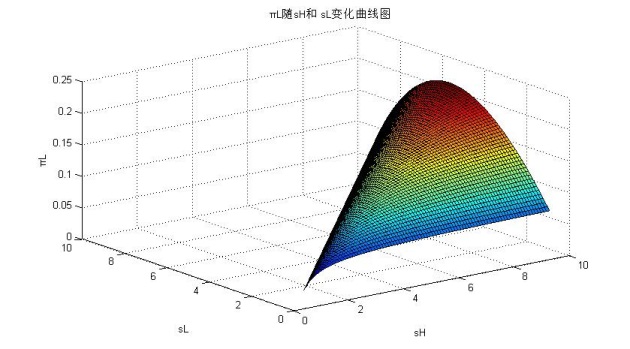


**S Fig. 1(left) Firm H’s profit changes with sH and sL. S Fig.1 (right) Firm L’s profit changes with sH and sl.**

**Appendix 2.**


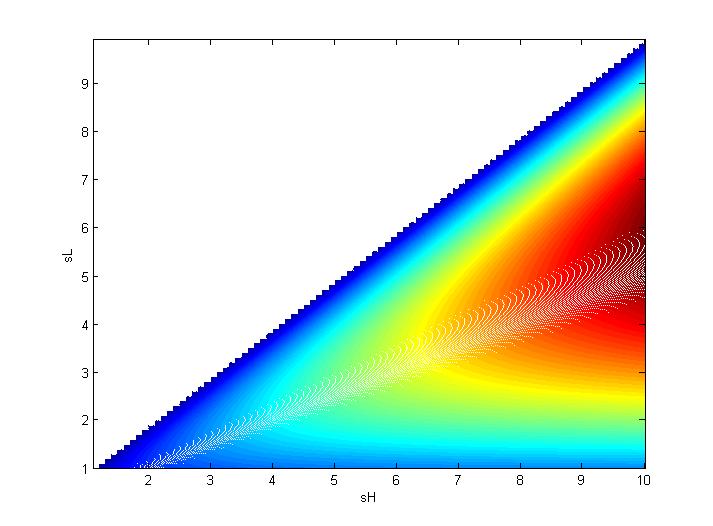


**S Fig.2 Contour map of firm L changes with sH and sL.**

Program of figure 1(left)：syms x y;

>> x=1:0.1:10;

>> y=1:0.1:10;

>> [x,y]=meshgrid(x,y);

>> idx=x-y<=0;

x(idx)=nan;

y(idx)=nan;

z=[4*(x.^2).*(x-y)]./(4*x-y).^2;

surf(x,y,z)

>> xlabel('sH');ylabel('sL');zlabel('πH');

>> title('πH changed with the variation of sH and sL ')

z2=[x.*y.*(x-y)]./(4*x-y).^2;

>> surf(x,y,z2)

>> xlabel('sH');ylabel('sL');z2label('πL')

xlabel('sH');ylabel('sL');zlabel('πL');

>> title('πL changed with the variation of sH and sL ')

**Appendix 3.**

When$a_{L}=0$, the firm H’s profit can be expressed as follows, therefore, $\pi_{H}^{*}=\frac{4(s_{H}+a_{H})^{2}(s_{H}+a_{H}-s_{L})}{\left[4(s_{H}+a_{H})-s_{L}\right]^{2}}-\frac{k}{2}a_{H}^{2}-\lambda$

That means, the following expression $\frac{\partial\pi_{H}^{*}}{\partial a_{H}}\left|a_{L}=0\right.>0$can be obtained only when

$$\frac{\left[8(s_{H}+a_{H})(s_{H}+a_{H}-s_{L})+(s_{H}+a_{H})^{2}\right]\left[4(s_{H}+a_{H})-s_{L}\right]-32(s_{H}+a_{H})^{2}(s_{H}+a_{H}-s_{L})}{a_{H}\left[4(s_{H}+a_{H})-s_{L}\right]^{3}}>k$$

When the following $a_{H}=0$ is satisfied, the firm L’s profit can be expressed as follows, therefore,

$$\pi_{L}^{*}=\frac{(s_{H}+a_{H})(s_{H}+a_{H}-s_{L}-a_{L})(s_{L}+a_{L})}{\left[4(s_{H}+a_{H})-(s_{L}+a_{L})\right]^{2}}-\frac{k}{2}a_{L}^{2}-\lambda$$

That means the following expression $\frac{\partial\pi_{L}^{*}}{\partial a_{L}}\left|a_{H}=0\right.>0$can be obtained only when

$$\frac{s_{H}(s_{H}-2s_{L}-2a_{L})\left[4s_{H}-(s_{L}+a_{L})\right]+2s_{H}(s_{H}-s_{L}-a_{L})(s_{L}+a_{L})}{a_{L}\left[4s_{H}-(s_{L}+a_{L})\right]^{3}}>k>0$$

**Appendix 4.**

According to the first condition on the changing qualities, we can obtain the following expressions.

$$\frac{\partial\pi_{H}^{M}}{\partial S_{H}}=\frac{\left[4S_{H}^{2}(S_{H}-S_{L})\right]'(4S_{H}-S_{L})^{2}-4S_{H}^{2}(S_{H}-S_{L})\left[(4S_{H}-S_{L})^{2}\right]'}{(4S_{H}-S_{L})^{4}}-K(S_{H}-s_{H})=0$$

$$\frac{\partial\pi_{L}^{*}}{\partial S_{L}}=\frac{\left[(S_{H}-S_{L})S_{H}S_{L}\right]'\left[(4S_{H}-S_{L})^{2}\right]-\left[(S_{H}-S_{L})S_{H}S_{L}\right]\left[(4S_{H}-S_{L})^{2}\right]'}{(4S_{H}-S_{L})^{4}}-K(S_{L}-s_{L})=0$$

That means the following equations can be obtained.

$$\frac{4S_{H}(4S_{H}^{2}-3S_{H}S_{L}+2S_{L}^{2})}{(4S_{H}-S_{L})^{3}}=ka_{H}$$

$$\frac{S_{H}^{2}(4S_{H}-7S_{L})}{(4S_{H}-S_{L})^{3}}=ka_{L}$$

$K(a_{H}-a_{L})=\frac{S_{H}(16S_{H}^{2}-12S_{H}S_{L}+8S_{L}^{2}-4S_{H}^{2}+7S_{H}S_{L})}{(4S_{H}-S_{L})^{3}}=\frac{S_{H}(12S_{H}^{2}-5S_{H}S_{L}+8S_{L}^{2})}{(4S_{H}-S_{L})^{3}}>0$.That means $a_{H}^{*}>a_{L}^{*}$.

Here, we consider that k=1, k=2, k=5, k=10;

With matlab 2013a, we set the following program.

>> syms sH aH sL aL

>>[aH,aL]=solve('4*(sH+aH)*(4*(sH+aH)^2-3*(sH+aH)*(sL+aL)+2*sL^2)/(4*(sH+aH)-(sL+aL))^3=aH','(sH+aH)^2*(4*(sH+aH)-7(sL+aL))/(4*(sH+aH)-(sL+aL))^3-aL','aH','aL')

As there is no analytic solution, we use *fsolve function* of matlab 2013 to solve the value solution of equations. Here, we set sH as $a$ which changes from 2 to 10 and sL as $b$ which changes from 1 to 10, k is the fixed advertising parameter. The specific function is shown as follows.

{ clear all

a=2:0.2:10;

b=1:0.2:10;

k=1:1:10;

x0=[0.3,0.02];

x=zeros(length(x0),length(a),10);

x_1=zeros(length(x0),length(a));

for k_iter=1:10

for n=1:length(a)

x0=fsolve(@(x)func(x,a(n),b(n),k(k_iter)),x0);

x_1(:,n)=x0;

end

x(:,:,k_iter)=x_1;

end

function F = func(x,a,b,k)

f1=4*(x(1)+a)*(4*(x(1)+a)^2-3*((x(1)+a)*(x(2)+b))+2*(x(2)+b)^2)/(4*(x(1)+a)-(x(2)+b))^3-x(1)*k;

f2=(x(1)+a)^2*(4*(x(1)+a)-7*(x(2)+b))/(4*(x(1)+a)-(x(2)+b))^3-x(2)*k;

F=[f1;f2];

}

5. {syms x y k a b

a=2.2;b=1.2;k=10;x=0.0286;y=0.0006;

f9=4*(x+a)^2*(x+a-y-b)/(4*(x+a)-(y+b))^2-0.5*k*x^2-0.01;

f10=(x+a)*(x+a-y-b)*(y+b)/(4*(x+a)-(y+b))^2-0.5*k*y^2-0.01;

f9

f10}

**S1 Table. The firm’s profit changes with variable parameters**

| $s_{H}$ | $s_{L}$ | $k$ | $\lambda$ | $a_{H}$ | $a_{L}$ | $\pi_{H}$ | $\pi_{L}$ |
| --- | --- | --- | --- | --- | --- | --- | --- |
| 2 | 1 | 1 | 0.01 | 0.2729 | 0.0192 | 0.3503 | 0.0344 |
| 2.2 | 1.2 | 1 | 0.01 | 0.2784 | 0.0133 | 0.3619 | 0.0402 |
| 2.4 | 1.4 | 1 | 0.01 | 0.2836 | 0.0078 | 0.3723 | 0.0454 |
| 2.6 | 1.6 | 1 | 0.01 | 0.2887 | 0.0028 | 0.3817 | 0.0501 |
| 2 | 1 | 2 | 0.01 | 0.1379 | 0.0080 | 0.3340 | 0.0327 |
| 2.2 | 1.2 | 2 | 0.01 | 0.1410 | 0.0047 | 0.3443 | 0.0381 |
| 2.4 | 1.4 | 2 | 0.01 | 0.1440 | 0.0017 | 0.3543 | 0.0429 |
| 2 | 1 | 3 | 0.01 | 0.0924 | 0.0049 | 0.3283 | 0.0321 |
| 2.2 | 1.2 | 3 | 0.01 | 0.0945 | 0.0026 | 0.3381 | 0.0374 |
| 2.4 | 1.4 | 3 | 0.01 | 0.0966 | 0.0006 | 0.3467 | 0.0420 |
| 2 | 1 | 4 | 0.01 | 0.0694 | 0.0035 | 0.3254 | 0.0318 |
| 2.2 | 1.2 | 4 | 0.01 | 0.0711 | 0.0018 | 0.3349 | 0.0370 |
| 2.4 | 1.4 | 4 | 0.01 | 0.0727 | 0.0002 | 0.3433 | 0.0415 |
| 2 | 1 | 5 | 0.01 | 0.0556 | 0.0027 | 0.3237 | 0.0316 |
| 2.2 | 1.2 | 5 | 0.01 | 0.0570 | 0.0013 | 0.3330 | 0.0367 |
| 2.4 | 1.4 | 5 | 0.01 | 0.0583 | 0.0001 | 0.3412 | 0.0412 |
| 2 | 1 | 6 | 0.01 | 0.0464 | 0.0022 | 0.3225 | 0.0315 |
| 2.2 | 1.2 | 6 | 0.01 | 0.0475 | 0.0010 | 0.3318 | 0.0366 |
| 2 | 1 | 7 | 0.01 | 0.0398 | 0.0019 | 0.3217 | 0.0314 |
| 2.2 | 1.2 | 7 | 0.01 | 0.0408 | 0.0009 | 0.3308 | 0.0364 |
| 2 | 1 | 8 | 0.01 | 0.0348 | 0.0016 | 0.3210 | 0.0313 |
| 2.2 | 1.2 | 8 | 0.01 | 0.0357 | 0.0007 | 0.3301 | 0.0364 |
| 2 | 1 | 9 | 0.01 | 0.0310 | 0.0014 | 0.3205 | 0.0313 |
| 2.2 | 1.2 | 9 | 0.01 | 0.0318 | 0.0006 | 0.3296 | 0.0363 |
| 2 | 1 | 10 | 0.01 | 0.0279 | 0.0013 | 0.3201 | 0.0312 |
| 2.2 | 1.2 | 10 | 0.01 | 0.0286 | 0.0006 | 0.3291 | 0.0362 |

**Appendix 5.**

$$\pi_{H}^{T}=\frac{(s_{H}+b_{H}-s_{L})\left[2(s_{H}+b_{H})-\lambda\right]^{2}}{\left[4(s_{H}+b_{H})-s_{L}\right]^{2}}-\frac{k}{2}b_{H}^{2}$$

$\frac{\partial\pi_{H}^{T}}{\partial b_{H}}\left|_{b_{H}=0}\right.=\frac{(2s_{H}-\lambda)\left[(6s_{H}-\lambda-s_{L})(4s_{H}-s_{L})-(4s_{H}-4s_{L})(4s_{H}-2\lambda)\right]}{(4s_{H}-s_{L})^{3}}$When $2s_{H}-\lambda>0$, as $2s_{H}+\lambda-s_{L}>0$, therefore, $6s_{H}-\lambda-s_{L}>4s_{H}-2\lambda$.$\frac{\partial\pi_{H}^{T}}{\partial b_{H}}\left|_{b_{H}=0}\right.>0$.

**Appendix 6.**

***Program for value solution of equations***

*When sH=2, sL=1, x=aH,y=aL, we can set the following program.*

{function q=myfun(p)

x=p(1);

y=p(2);

q(1)=(4*(2+x)*(4*(2+x)^2-3*(2+x)*(1+y)+2*(1+y)^2)/(4*(2+x)-1-y)^3)-x;

q(2)=((2+x)^2*(4*(2+x)-7*(1+y))/(4*(2+x)-1-y)^3)-y;

x=fsolve('myfun',[0.5,0.5],optimset('Display','off'))}

$$\pi_{L}^{T}=\frac{s_{H}\left[s_{H}-(s_{L}+b_{L})\right]\left[(s_{L}+b_{L})-2\lambda\right]^{2}}{\left[4s_{H}-(s_{L}+b_{L})\right]^{2}(s_{L}+b_{L})}-\frac{k}{2}b_{L}^{2}$$

$\frac{\partial\pi_{L}^{T}}{\partial b_{L}}\left|_{b_{L}=0}\right.=\frac{s_{H}(s_{L}-2\lambda)(2s_{H}-3s_{L}+2\lambda)(4s_{H}-s_{L})s_{L}-s_{H}(s_{H}-s_{L})(s_{L}-2\lambda)^{2}(4s_{H}-3s_{L})}{(4s_{H}-s_{L})s_{L}^{2}}$ When $(2s_{H}-3s_{L}+2\lambda)(4s_{H}-s_{L})s_{L}>(s_{H}-s_{L})(s_{L}-2\lambda)(4s_{H}-3s_{L})$,$\frac{\partial\pi_{L}^{T}}{\partial b_{L}}\left|_{b_{L}=0}\right.>0$. That means$2s_{H}-3s_{L}+2\lambda>s_{H}-s_{L}$, and then $s_{H}>2(s_{L}-\lambda)$ and $s_{L}>2\lambda$, which means as long as $s_{H}>s_{L}>2\lambda$, $\frac{\partial\pi_{L}^{T}}{\partial b_{L}}\left|_{b_{L}=0}\right.>0$.

**Appendix 7.**

$$\pi_{H}^{T}=\frac{(S_{H}^{T}-S_{L}^{T})(2S_{H}^{T}-\lambda)^{2}}{(4S_{H}^{T}-S_{L}^{T})^{2}}-A_{H}^{T}$$

$$\pi_{L}^{T}=\frac{S_{H}^{T}(S_{H}^{T}-S_{L}^{T})(S_{L}^{T}-2\lambda)^{2}}{(4S_{H}^{T}-S_{L}^{T})^{2}S_{L}^{T}}-A_{L}^{T}$$

According to the first condition on *S_H_*, the following expression are satisfied.

$\begin{matrix} \frac{\partial\pi_{H}}{\partial S_{H}}=\frac{\left[(S_{H}^{T}-S_{L}^{T})(2S_{H}^{T}-\lambda)^{2}\right]'(4S_{H}^{T}-S_{L}^{T})^{2}-(S_{H}^{T}-S_{L}^{T})(2S_{H}^{T}-\lambda)^{2}\left[(4S_{H}^{T}-S_{L}^{T})^{2}\right]'}{(4S_{H}^{T}-S_{L}^{T})^{4}}-kb_{H} \\ =\frac{4S_{H}^{T}(4S_{H}^{T2}-3S_{H}^{T}S_{L}^{T}+2S_{L}^{T2})-8S_{H}^{T}S_{L}^{T}\lambda-4\lambda S_{L}^{T2}-\lambda^{2}(4S_{H}^{T}-7S_{L}^{T})}{(4S_{H}^{T}-S_{L}^{T})^{3}}-kb_{H}=0 \end{matrix}$Therefore, $\frac{4S_{H}^{T}(4S_{H}^{T2}-3S_{H}^{T}S_{L}^{T}+2S_{L}^{T2})}{(4S_{H}^{T}-S_{L}^{T})^{3}}-\frac{8S_{H}^{T}S_{L}^{T}\lambda+4\lambda S_{L}^{T2}+\lambda^{2}(4S_{H}^{T}-7S_{L}^{T})}{(4S_{H}^{T}-S_{L}^{T})^{3}}=kb_{H}$

$$\frac{\partial\pi_{L}^{T}}{\partial S_{L}^{T}}=S_{H}^{T}\frac{\left[(S_{H}^{T}-S_{L}^{T})(S_{L}^{T}-2\lambda)^{2}\right]'(4S_{H}^{T}-S_{L}^{T})^{2}S_{L}^{T}-(S_{H}^{T}-S_{L}^{T})(S_{L}^{T}-2\lambda)^{2}\left[(4S_{H}^{T}-S_{L}^{T})^{2}S_{L}^{T}\right]'}{\left[(4S_{H}^{T}-S_{L}^{T})^{2}S_{L}^{T}\right]^{2}}$$

$$=S_{H}^{T}\frac{4S_{H}^{T2}S_{L}^{T2}-7S_{L}^{T3}S_{H}^{T}+4\lambda(S_{L}^{T}-\lambda)(4S_{H}^{T2}-3S_{H}^{T}S_{L}^{T}+2S_{L}^{T2})}{(4S_{H}^{T}-S_{L}^{T})^{3}S_{L}^{T2}}-kb_{L}=0$$

Therefore, $\frac{S_{H}^{T2}(4S_{H}^{T}-7S_{L}^{T})}{(4S_{H}^{T}-S_{L}^{T})^{3}}+\frac{4\lambda(S_{L}^{T}-\lambda)(4S_{H}^{T2}-3S_{H}^{T}S_{L}^{T}+2S_{L}^{T2})}{(4S_{H}^{T}-S_{L}^{T})^{3}S_{L}^{T2}}=kb_{L}$

**Appendix 8.**

*When sH=2, sL=1, x=bH,y=bL,k=1,*$\lambda=0.1$*we can set the following program to explore the aforementioned equations .*

{ function q=myfun7(p)

x=p(1);

y=p(2);

q(1)=(4*(2+x)*(4*(2+x)^2-3*(2+x)*(1+y)+2*(1+y)^2)-8*(2+x)*(1+y)*0.1-4*0.1*(1+y)^2-0.1^2*(4*(2+x)-7*(1+y)))/(4*(2+x)-1-y)^3-x;

q(2)=(2+x)^2*(4*(2+x)-7*(1+y))/(4*(2+x)-1-y)^3+4*0.1*(1+y-0.1)*(4*(2+x)^2-3*(2+x)*(1+y)+2*(1+y)^2)/((4*(2+x)-1-y)^3*(1+y)^2)-y;

x=fsolve('myfun7',[0.5,0.5],optimset('Display','off'))}

Then we can obtain the results of both firms’ profits.

{ syms x y

X= 0.2691;y=0.0291

z=(2+x-1-y)*(2*(2+x)-0.1)^2/(4*(x+2)-(y+1))^2-0.5*x^2

w=(2+x)*((2+x)-(1+y))*((1+y)-2*0.1)^2/((4*(2+x)-(1+y))^2*(1+y))-0.5*y^2}

According to the equations, we reset the program to solve the equations when the parameter is variable.

{ clear all

a=2:0.2:10;

b=1:0.2:10;

k=[1 2 5 10];

u=[0.1 0.2 0.3]

x0=[0.3,0.02];

x=zeros(length(x0),length(a),4,3);

x_1=zeros(length(x0),length(a));

for k_iter=1:4

for u_iter=1:3

for n=1:length(a)

x0=fsolve(@(x)func2(x,a(n),b(n),k(k_iter),u(u_iter)),x0);

x_1(:,n)=x0;

end

end

x(:,:,k_iter,u_iter)=x_1;

end

function F = func2(x,a,b,k,u)

f1=(4*(x(1)+a)*(4*(x(1)+a)^2-3*((x(1)+a)*(x(2)+b))+2*(x(2)+b)^2)-8*(x(1)+a)*(x(2)+b)*u-4*u*(x(2)+b)^2-u^2*(4*(x(1)+1)-7*(x(2)+b)))/(4*(x(1)+a)-(x(2)+b))^3-x(1)*k;

f2=(x(1)+a)^2*(4*(x(1)+a)-7*(x(2)+b))/(4*(x(1)+a)-(x(2)+b))^3+(4*u*((x(2)+b)-u)*((4*(x(1)+b))^2-3*((x(1)+a)*(x(2)+b)+2*(x(2)+b)^2)))/((4*(x(1)+a)-(x(2)+b))^3*(x(2)+b)^2)-x(2)*k;

F=[f1;f2];

}
